# Comparative RNA-sequencing-based transcriptome profiling of buds from profusely flowering ‘Qinguan’ and weakly flowering ‘Nagafu no. 2’ apple varieties reveals novel insights into the regulatory mechanisms underlying floral induction

**DOI:** 10.1186/s12870-018-1555-3

**Published:** 2018-12-22

**Authors:** Xilong Chen, Siyan Qi, Dong Zhang, Youmei Li, Na An, Caiping Zhao, Juan Zhao, Kamran Shah, Mingyu Han, Libo Xing

**Affiliations:** 1 College of Horticulture, Northwest Agriculture & Forestry University, Yangling, 712100 Shaanxi China; 2 College of Mechanical and Electronic Engineering, Northwest Agriculture & Forestry University, Yangling, 712100 Shaanxi China

**Keywords:** Apple trees, Carbon, Fatty acids, Hormones, Floral induction, RNA-seq

## Abstract

**Background:**

Floral induction is an important stage in the apple tree life cycle. In ‘Nagafu No. 2’, which was derived from a ‘Fuji’ bud sport, flower bud formation is associated with serious problems, such as fewer and inferior flower buds, a long juvenile phase, and an alternate bearing phenotype. Moreover, the molecular regulatory mechanisms underlying apple floral induction remain unknown. To characterize these mechanisms, we compared the RNA-sequencing-based transcriptome profiles of buds during floral induction in profusely flowering ‘Qinguan’ and weakly flowering ‘Nagafu No. 2’ apple varieties.

**Results:**

Genes differentially expressed between the buds of the two varieties were mainly related to carbohydrate, fatty acid, and lipid pathways. Additionally, the steady up-regulated expression of genes related to the fatty acid and lipid pathways and the down-regulated expression of starch synthesis-related genes in the carbon metabolic pathway of ‘Qinguan’ relative to ‘Nagafu No. 2’ were observed to contribute to the higher flowering rate of ‘Qinguan’. Additionally, global gene expression profiling revealed that genes related to cytokinin, indole-3-acetic acid, and gibberellin synthesis, signalling, and responses (i.e., factors contributing to cell division and differentiation and bud growth) were significantly differentially expressed between the two varieties. The up-regulated expression of genes involved in abscisic acid and salicylic acid biosynthesis via shikimate pathways as well as jasmonic acid production through fatty acid pathways in ‘Qinguan’ buds were also revealed to contribute to the floral induction and relatively high flowering rate of this variety. The differential expression of transcription factor genes (i.e., *SPL*, *bZIP*, *IDD*, and *MYB* genes) involved in multiple biological processes was also observed to play key roles in floral induction. Finally, important flowering genes (i.e., *FT*, *FD*, and *AFL*) were significantly more highly expressed in ‘Qinguan’ buds than in ‘Nagafu No. 2’ buds during floral induction.

**Conclusions:**

A complex genetic network of regulatory mechanisms involving carbohydrate, fatty acid, lipid, and hormone pathways may mediate the induction of apple tree flowering.

**Electronic supplementary material:**

The online version of this article (10.1186/s12870-018-1555-3) contains supplementary material, which is available to authorized users.

## Background

Apple (*Malus domestica* Borkh*.*) is an economically important fruit tree species worldwide [1]. The flowering characteristics of apple cultivars vary widely. For example, ‘Nagafu No. 2’, a dominant variety representing 65% of the total cultivated apple area in China [2, 3], exhibits serious problems regarding floral induction and formation, such as a long juvenile phase, alternate bearing phenotype, and low flower and fruit production. In contrast, ‘Qinguan’, an elite variety bred in China, has a strong flowering ability, high yields, and strong disease resistance [4]. Consequently, a comprehensive characterization of the physiological and molecular regulatory mechanisms underlying floral induction and bud formation in different apple varieties is very important for solving problems with flowering.

Floral induction is an important stage in the plant life cycle that is regulated by complex networks involving multiple environmental and internal signals to ensure the appropriate timing of flowering [5]. Six major flowering pathways are associated with the floral induction regulatory process, namely, vernalization, autonomous, photoperiod, gibberellic acid (GA), thermosensory, and aging pathways [6, 7]. Additionally, studies have revealed that key flowering genes (*FT, SOC1, LEAF, SPLs,* and *AP1*) involved in multiple flowering pathways play an important role in floral induction [6, 8, 9].

Carbohydrates (e.g., sucrose, glucose, and starch) have important signalling and energy roles in floral induction across multiple flowering pathways [9, 10]. Trehalose-6-phosphate (T6P), a key sugar signalling substance found mainly in the shoot apical meristem and leaves, exhibits age-dependent responses to environmental stresses and carbohydrate levels to promote flowering [9]. Additionally, sugar signals can link to flowering pathways associated with the regulation of transcription factor (TF) AtIDD8 in photoperiodic flowering [11]. Other studies have indicated that carbon and lipid metabolic substances [(i.e. fatty acids and phosphatidylcholine (PC)] are also important for regulating floral induction in plants [12, 13].

Plant hormones regulate multiple floral induction-related pathways, such as GA [8], autonomous [14], and photoperiod [15] pathways, in addition to stress responses [16]. The stress-related hormone abscisic acid (ABA) regulates flowering mainly via photoperiodic and sugar signalling pathways [17, 18]. Additionally, previous studies proved that the application of exogenous cytokinin (CTK) can promote flowering in woody plants [19] and that CTK regulates flowering directly by affecting the expression of floral-related genes (i.e., *FT* and *SOC1*) [6, 20]. Moreover, GA has positive and negative regulatory roles, respectively, in woody [21, 22] and model annual plants [23]. Other plant hormones, such as salicylic acid (SA) and jasmonic acid (JA), have key functions related to flowering and are involved in multiple biological processes [16, 24].

While the regulation of flowering in plants exposed to various environmental stresses has been studied for a long time, the molecular regulatory mechanisms of floral induction in woody fruit trees remain unknown. In this study, we applied RNA sequencing (RNA-seq) on the Illumina platform to compare gene expression patterns between the buds of profusely flowering ‘Qinguan’ and weakly flowering ‘Nagafu No. 2’ apple varieties during growth and floral induction. We observed that a complex genetic network of carbon, fatty acid, lipid, and hormone-associated signalling regulatory mechanisms mediates apple tree floral induction. We also analysed sugar-, hormone-, and flowering-related gene expression patterns in the buds of both apple varieties in a quantitative real-time polymerase chain reaction (qRT-PCR) assay. Our findings may be useful for further characterizing the molecular regulatory mechanisms underlying floral induction in apple trees.

## Results

### Dynamic changes in shoot and bud growth, flowering and bud break rates, and branch type in ‘Qinguan’ and ‘Nagafu No. 2’ apple varieties

Shoot lengths of ‘Nagafu No. 2’ increased gradually from 0 to 28 days after full bloom (DAFB) and exhibited peak increases at 14 DAFB, with no further changes observed from 28 to 42 DAFB (Additional file 1: Figure S1). In ‘Qinguan’ trees, the peak shoot length increase occurred at 7 DAFB, and shoot lengths were significantly lower than those of ‘Nagafu No. 2’ from 14 to 42 DAFB (Additional file 1: Figure S1). During the 3-year study period (2013–2015), the proportion of spur shoots was significantly higher in ‘Qinguan’ than in ‘Nagafu No. 2’, while the opposite was true for long shoots (Additional file 1: Figure S2). Although no significant difference in the proportion of intermediate shoots was observed between the two varieties in 2013 and 2014, this value was higher in ‘Qinguan’ than in ‘Nagafu No. 2’ in 2015 (Additional file 1: Figure S2).

**Fig. 1:**
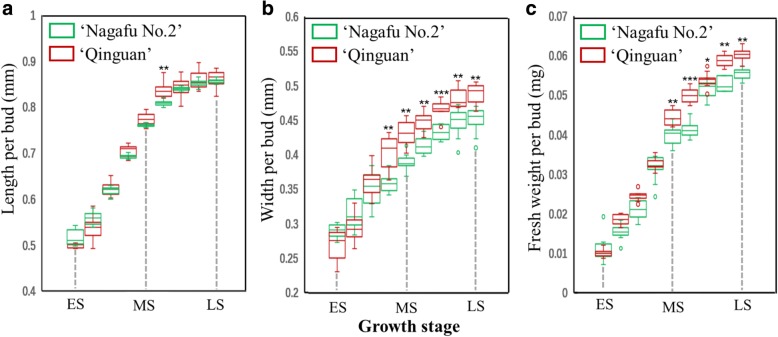
Bud growth during floral induction in ‘Qinguan’ and ‘Nagafu No. 2’ apple varieties. **a** Length. **b** Width. **c** Fresh weight. ES, MS, and LS correspond to the early, middle, and late stages of flower bud differentiation, respectively. Data are presented as the mean ± standard error, *n* = 12. *p < 0.05; **p < 0.01; ***p < 0.001; ns, non-significant (*p* > 0.05)

No significant differences in bud length were detected between ‘Qinguan’ and ‘Nagafu No. 2’ from the early stage (ES; 5 May 2013) to the late stage (LS; 25 June 2013) of flower bud physiological differentiation (Fig. 1a), whereas bud width was significantly higher in ‘Qinguan’ than in ‘Nagafu No. 2’ trees during the later stages of floral induction [roughly the middle stage (MS; 1 June 2013) to the LS] (Fig. 1b). Moreover, bud fresh weights from the MS to the LS were significantly higher in ‘Qinguan’ than in ‘Nagafu No. 2’ (Fig. 1c).

**Fig. 2:**
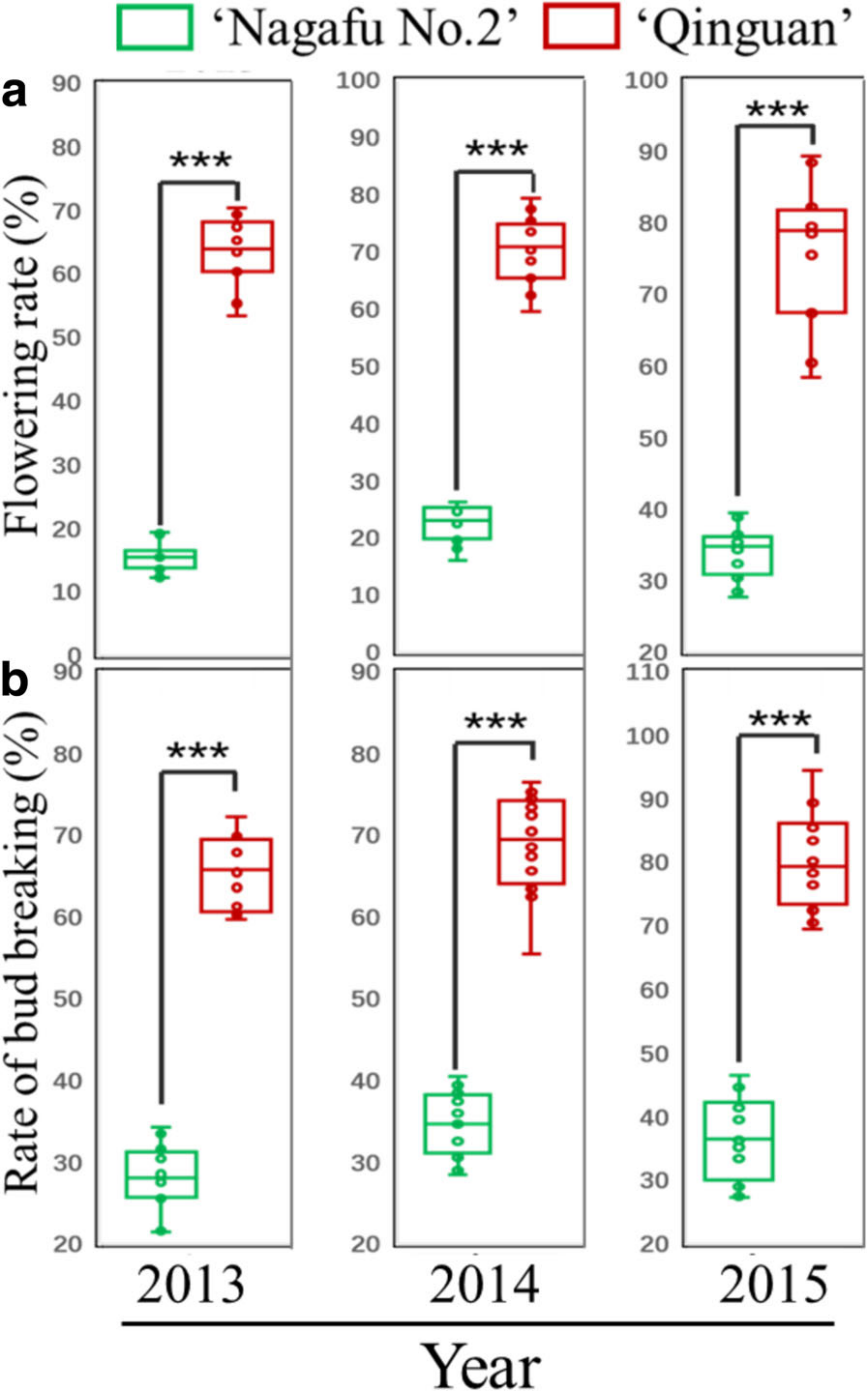
Flowering and bud break rates in ‘Qinguan’ and ‘Nagafu No. 2’ apple varieties. **a** Flowering rates on 12 April 2013, 2014, and 2015. **b** Bud break rates on 10 March 2013, 2014, and 2015. Data are presented as the mean ± standard error, *n* = 12. *p < 0.05; **p < 0.01; ***p < 0.001; ns, non-significant (*p* > 0.05)

In March 2013, 2014, and 2015, bud break rates were significantly higher in ‘Qinguan’ than in ‘Nagafu No. 2’ (Fig. 2). According to the statistical analysis, flowering rates were also significantly higher in ‘Qinguan’ than in ‘Nagafu No. 2’ from 2013 to 2015 (Fig. 2).

### Dynamic changes in sugar and hormone levels in buds during floral induction in the ‘Qinguan’ and ‘Nagafu No. 2’ apple varieties

**Fig. 3:**
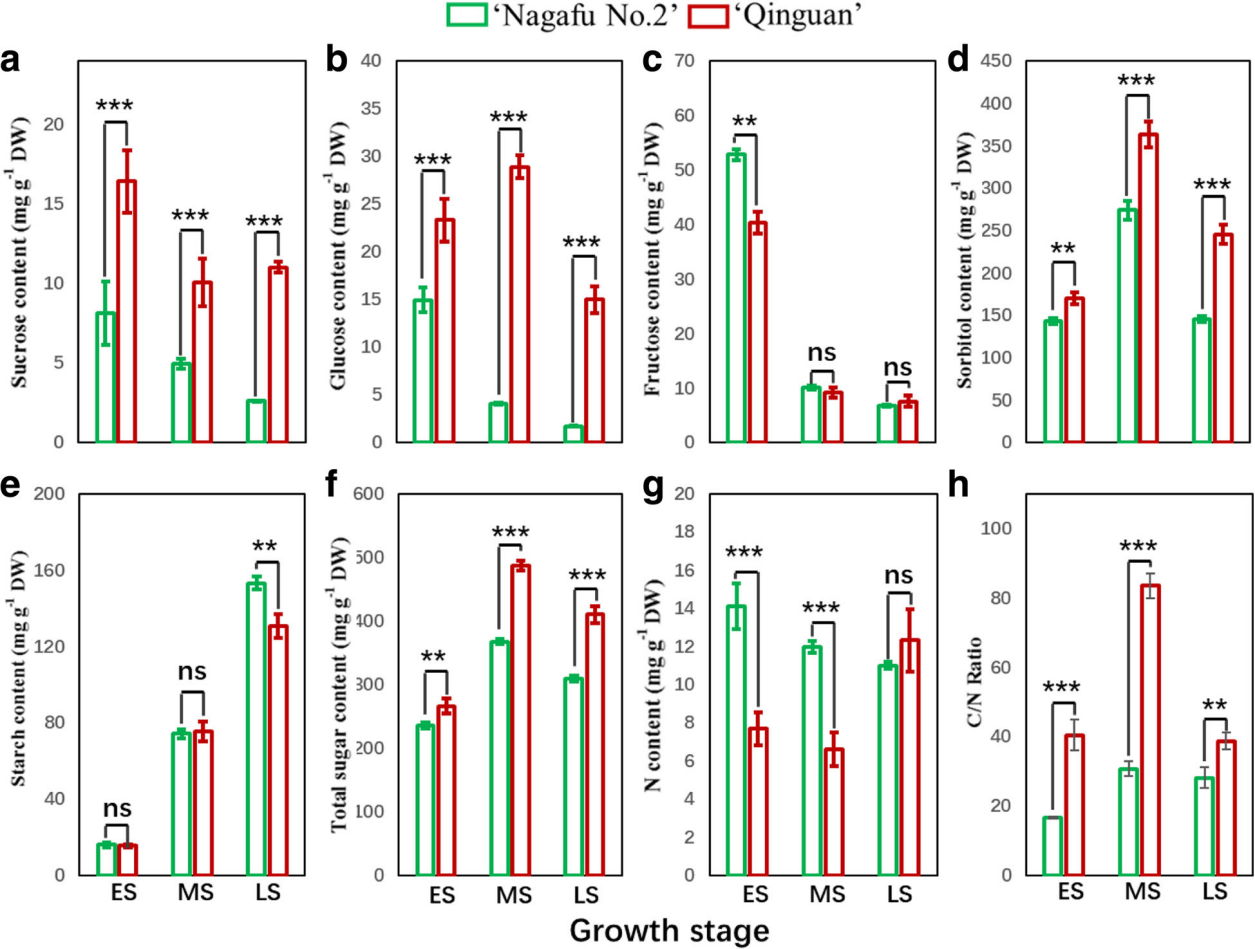
Nitrogen and sugar contents and the ratio of carbon to nitrogen in the buds of ‘Qinguan’ and ‘Nagafu No. 2’ apple varieties during the flower bud physiological differentiation stage. **a** Sucrose. **b** Glucose. **c** Fructose. **d** Sorbitol. **e** Starch. **f** Total sugar. **g** Nitrogen. **h** C/N. N%: percentage of nitrogen content; C/N: carbon to nitrogen ratio. ES, MS, and LS correspond to the early, middle, and late stages of flower bud differentiation, respectively. Data are presented as the mean ± standard error, *n* = 3. *p < 0.05; **p < 0.01; ***p < 0.001; ns, non-significant (*p* > 0.05)

Sucrose, glucose, sorbitol, and total sugar contents of buds during floral induction (ES to LS) were significantly higher in ‘Qinguan’ than in ‘Nagafu No. 2’ (Fig. 3), whereas bud fructose levels during the ES and starch contents during the LS were higher in ‘Nagafu No. 2’ (Fig. 3). Additionally, the N content of buds in the ES and MS of floral induction was significantly lower in ‘Qinguan’ than in ‘Nagafu No. 2’ (Fig. 3). In contrast, the C/N ratio of ‘Qinguan’ buds was significantly higher than that of ‘Nagafu No. 2’ buds from the ES to the LS of floral induction (Fig. 3).

**Fig. 4:**
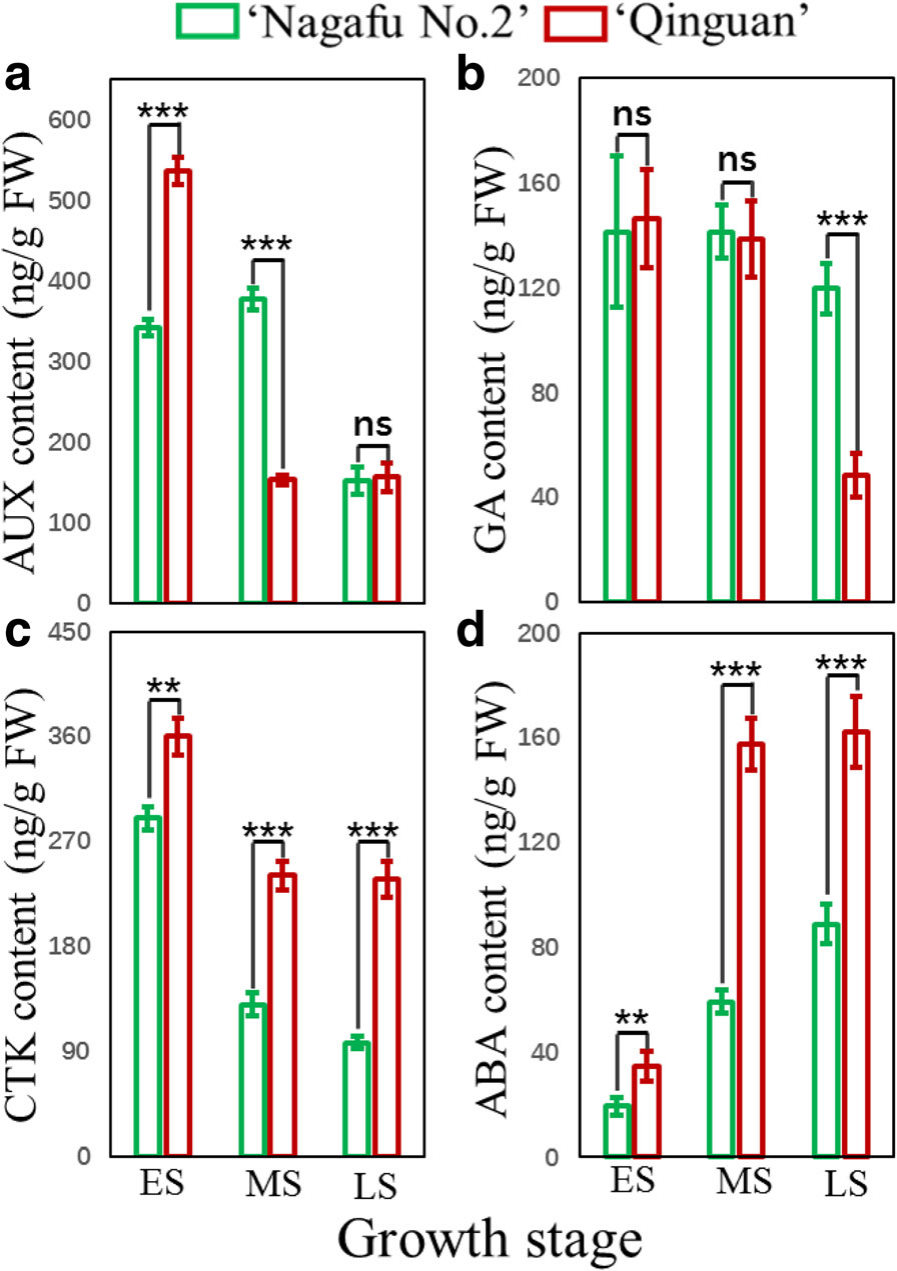
Hormone contents of buds during the flower bud physiological differentiation stage in ‘Qinguan’ and ‘Nagafu No. 2’ apple varieties. **a** Auxin (AUX). **b** Cytokinin (CTK). **c** Gibberellin (GA). **d** Abscisic acid (ABA). ES, MS, and LS correspond to the early, middle, and late stages of flower bud differentiation, respectively. Data are presented as the mean ± standard error, *n* = 3. *p < 0.05; **p < 0.01; ***p < 0.001; ns, non-significant (*p* > 0.05)

During the ES, the bud auxin content was significantly higher in ‘Qinguan’ than in ‘Nagafu No. 2’, with the opposite pattern observed during the MS of floral induction (Fig. 4a). The bud GA content was significantly lower in ‘Qinguan’ than in ‘Nagafu No. 2’ only during the LS (Fig. 4b). Moreover, the ABA and CTK contents were significantly higher in ‘Qinguan’ buds than in ‘Nagafu No. 2’ buds during the entire bud physiological differentiation period (ES to LS) (Fig. 4c and d).

### Sequencing and global analysis of bud transcriptomes of apple varieties ‘Qinguan’ and ‘Nagafu No. 2’ during floral induction

Six separate bud RNA-seq libraries were constructed and sequenced for ‘Qinguan’ and ‘Nagafu No. 2’ at different bud growth stages (ES, MS, and LS). Details of the sequencing data for each sample are given in Additional file 1: Tables S1 and S2. The distribution of sample read densities on chromosomes is provided in Additional file 1: Figure S3. The expression levels of 1151 (QE_FE), 1114 (QM_FM), and 1440 (QL_FL) differentially expressed genes (DEGs) were up-regulated in ‘Qinguan’ buds relative to ‘Nagafu No. 2’ buds during floral induction (Additional file 1: Figure S4), whereas 1043 (QE_FE), 1025 (QM_FM), and 1285 (QL_FL) DEGs were down-regulated (Additional file 1: Figure S4).

**Fig. 5:**
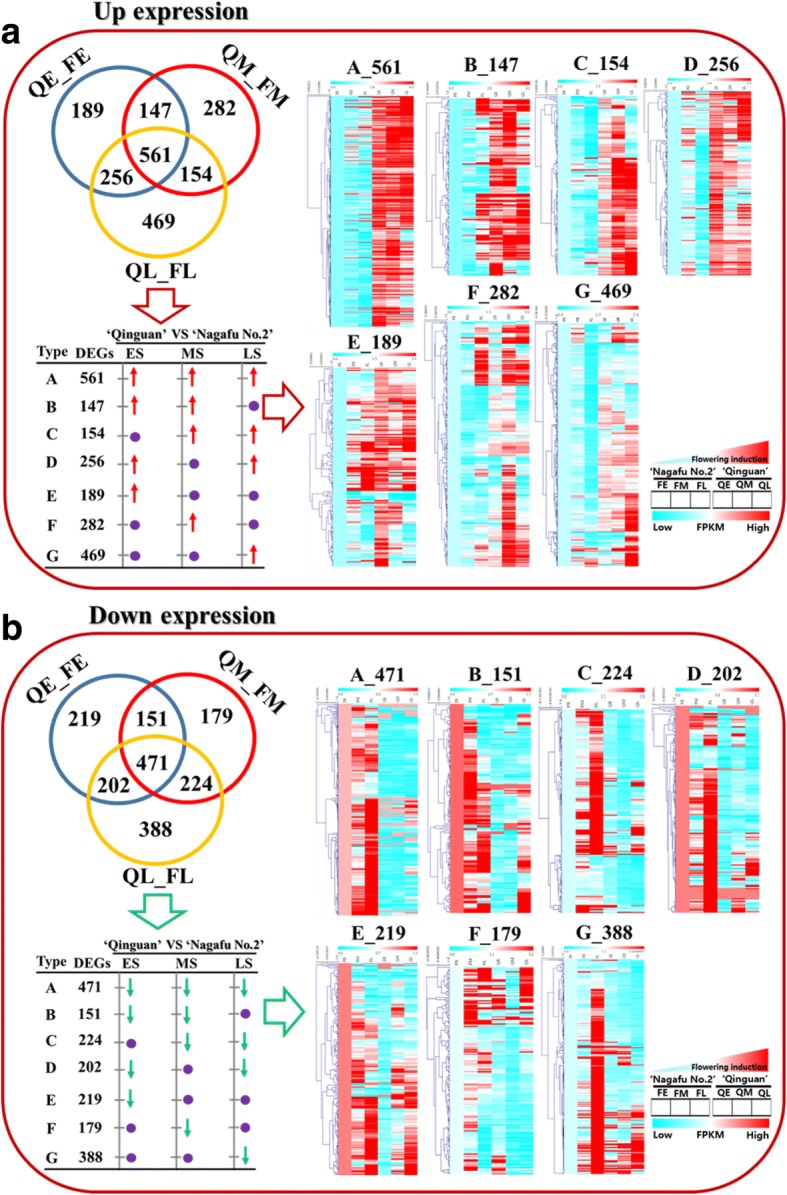
Global analysis of bud transcriptomes during floral induction in ‘Qinguan’ and ‘Nagafu No. 2’ apple varieties. **a** Differentially expressed genes (DEGs) up-regulated in ‘Qinguan’ buds compared with ‘Nagafu No. 2’ buds during floral induction. **b** DEGs down-regulated in ‘Qinguan’ buds compared with ‘Nagafu No. 2’ buds during floral induction. Venn diagrams and the results of a cluster analysis of seven expression pattern types (a-, b-, c-, d-, e-, f-, and g-type) of up- and down-regulated DEGs between ‘Qinguan’ and ‘Nagafu No. 2’ are shown. QE_FE, QM_FM, and QL_FL correspond to the early, middle, and late stages of flower bud differentiation, respectively. The FPKM values were used for the cluster analysis

Venn diagram and cluster analyses allowed us to categorize the up- and down-regulated DEGs of ‘Qinguan’ and ‘Nagafu No. 2’ buds into the following seven expression pattern groups: a-type, b-type, c-type, d-type, e-type, f-type, and g-type (Fig. 5a). Additionally, 561 DEGs that were up-regulated in ‘Qinguan’ relative to ‘Nagafu No. 2’ exhibited an a-type pattern (i.e., significantly higher expression level during the ES, MS, and LS of floral induction) (Fig. 5a). There were 147, 154, and 256 DEGs that were more highly expressed in ‘Qinguan’ during the ES and MS (b-type), the MS and LS (c-type), and the ES and LS (d-type), respectively (Fig. 5a). Furthermore, 189, 282, and 469 DEGs were detected belonging to the e-type, f-type, and g-type groups, respectively, corresponding in turn to significantly higher expression levels during the ES, MS, and LS (Fig. 5a).

With respect to DEGs down-regulated in ‘Qinguan’ relative to ‘Nagafu No. 2’, 471 were a-type genes, with significantly lower expression levels in the ES, MS, and LS of floral induction (Fig. 5b), while 151, 224, and 202 DEGs belonging to the b-type, c-type, and d-type groups had significantly lower expression levels during the ES and MS, the MS and LS, and the ES and LS, respectively (Fig. 5b). Finally, 219, 179, and 388 DEGs exhibited e-type, f-type, and g-type expression patterns, with significantly lower expression levels during the ES, MS, and LS, respectively (Fig. 5b).

**Fig. 6:**
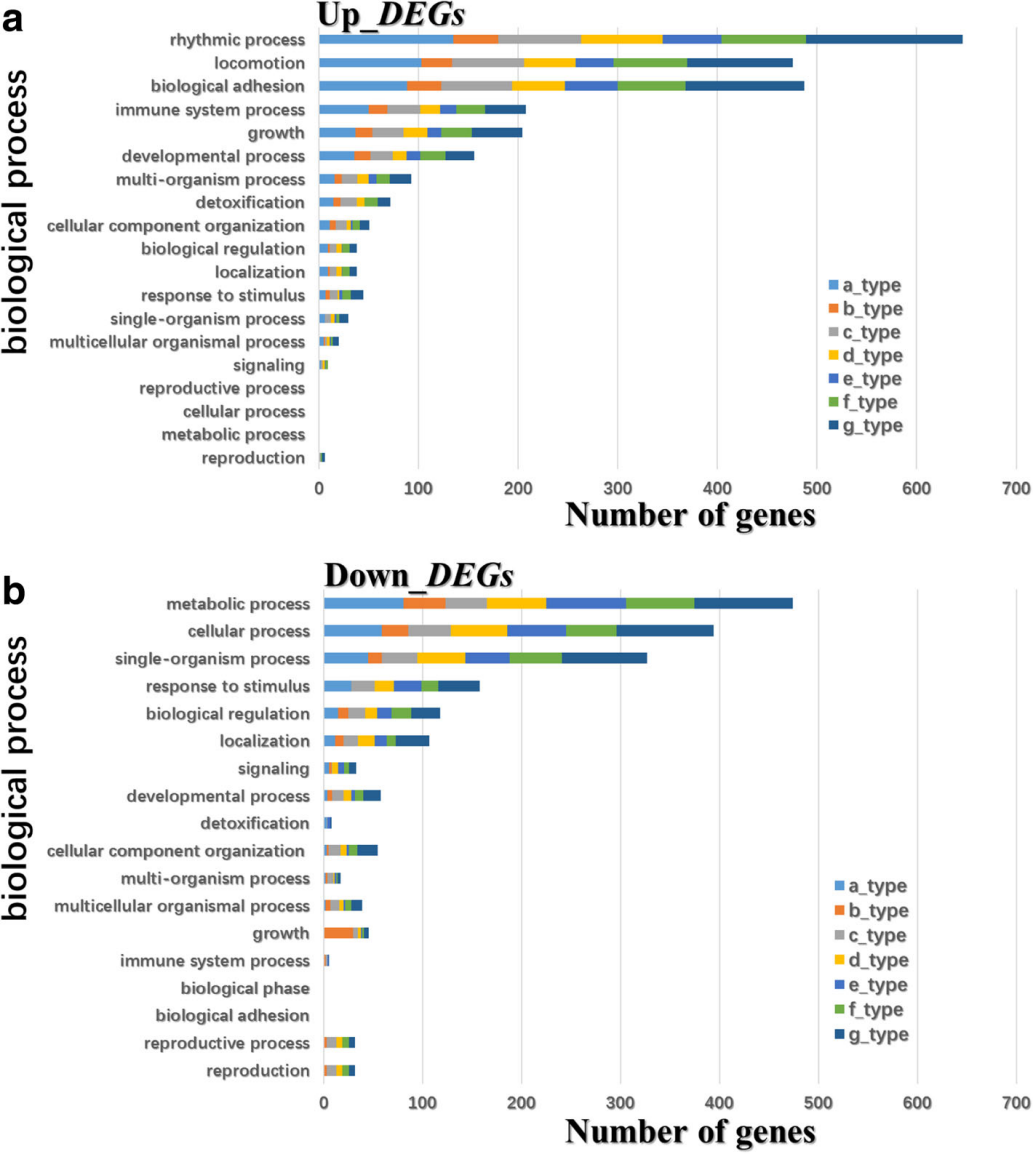
Number of differentially expressed genes (DEGs) between ‘Qinguan’ and ‘Nagafu No. 2’ involved in biological processes during floral induction. **a** DEGs up-regulated in ‘Qinguan’ buds relative to ‘Nagafu No. 2’ buds. **b** DEGs down-regulated in ‘Qinguan’ buds relative to ‘Nagafu No. 2’ buds. The seven types of DEG expression patterns (a-, b-, c-, d-, e-, f-, and g-type) are the same as those in the cluster analysis in Fig. 7

To identify biological processes, cellular components, and molecular functions enriched in apple buds during floral induction, we performed a gene ontology (GO) functional analysis of the above-mentioned up- and down-regulated DEGs (Fig. 6, Additional file 1: Figures S5, and S6). With respect to DEGs up-regulated in ‘Qinguan’ relative to ‘Nagafu No. 2’, rhythmic process, locomotion, biological adhesion, and immune system process were the four most highly enriched biological process categories (Fig. 6), whereas metabolic, cellular, single-organism process, and response to stimulus were the four most heavily represented biological process categories among DEGs down-regulated in ‘Qinguan’ compared with ‘Nagafu No. 2’ (Fig. 6). Additionally, detailed information regarding the Kyoto Encyclopedia of Genes and Genomes (KEGG) analysis of up- and down-regulated genes, including the seven expression pattern types (a-, b-, c-, d-, e-, f-, and g-type), in ‘Qinguan’ buds relative to ‘Nagafu No. 2’ buds is provided in Additional file 2.

**Fig. 7:**
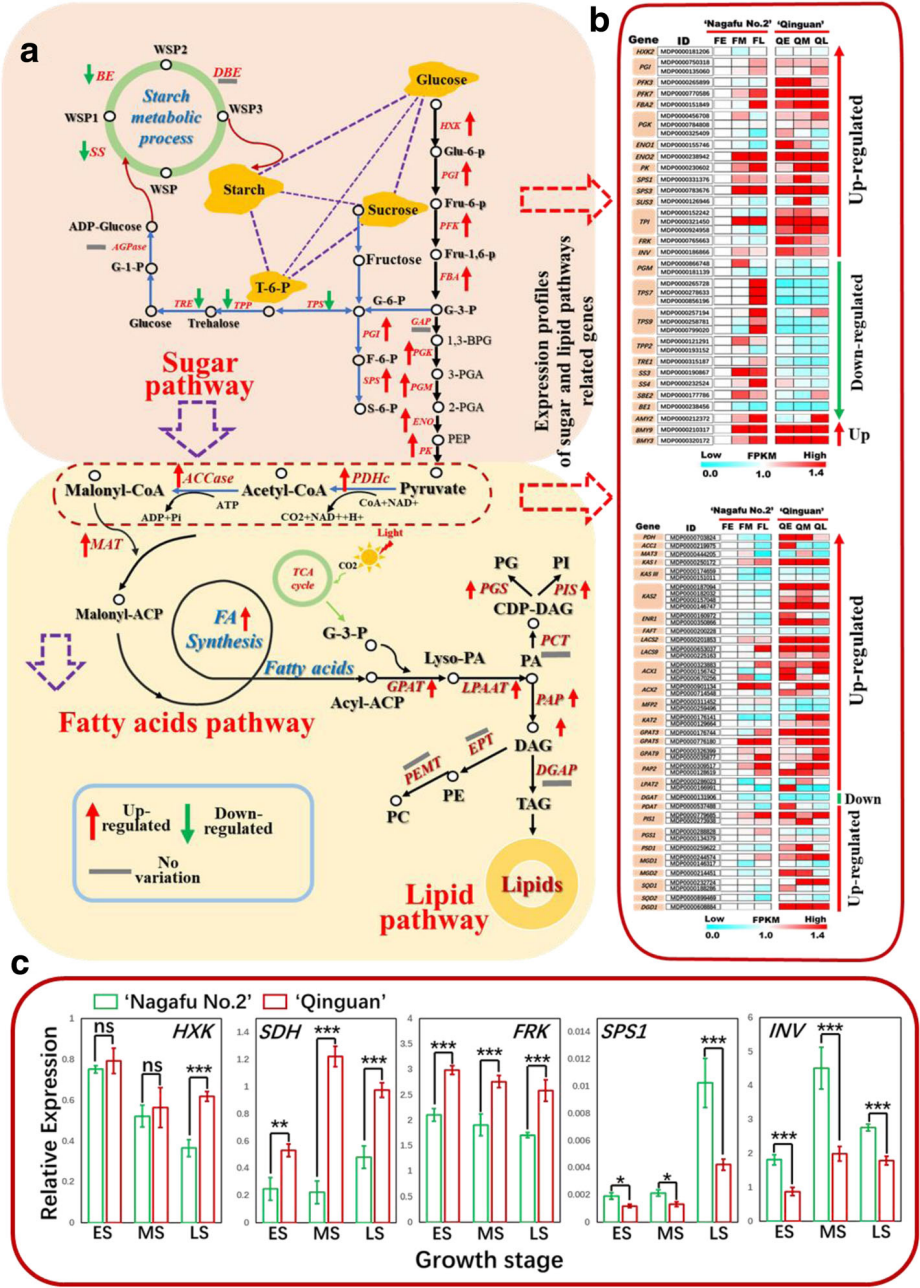
Differential expression of genes centred around specialized metabolic pathways (carbohydrate, fatty acid, and lipid) in ‘Qinguan’ and ‘Nagafu No. 2’ buds. **a** Model of carbohydrate, fatty acid, and lipid metabolism and regulation in the two apple varieties as reconstructed from transcriptomic evidence. Expression trends are represented by arrows. **b** Expression profiles of differentially expressed genes (DEGs) involved in carbohydrate, fatty acid, and lipid pathways according to RNA-sequencing and (**c**) quantitative real-time PCR. Abbreviations in (A) are as follows: G-1-P, glucose-1-phosphate; TP, triosephosphate; G-6-P, glucose-6-phosphate; S-6-P, sorbitol-6-phosphate; F-6-P, fructose-6-phosphate; S-6-P, sucrose-6-phosphate; UDPG, UDP-glucose; ADPG, ADP-glucose; PGM, phosphoglucomutase; SUS, sucrose synthase; HXK, hexokinase; PGI, phosphoglucose isomerase; FRK, fructokinase; UGP, UDP-glucose pyrophosphorylase; AGP, ADP-glucose pyrophosphorylase; SPS, sucrose phosphate synthase; SPP, sucrose phosphate phosphatase; AMY, α-amylase; BAM, β-amylase; A/N-INV, alkaline/neutral invertase; TPS, trehalose-6-phosphate synthase; T6P, trehalose-6-phosphate; SBE, starch branching enzymes; SS, starch synthase; TPP, trehalose-6-phosphatase; TRE, trehalase; SP, starch phosphorylase; DAG, diacylglycerol; DHAP, dihydroxyacetone phosphate; Gly3P, glyceraldehyde-3-phosphate; G3P, glycerol-3-phosphate; GL, galactoglycerolipids; Lyso-PA, lyso-phosphatidic acid; Lyso-PL, lyso-phospholipids; PA, phosphatidic acid; PE, phosphatidyl-ethanolamine; PG, phosphatidylglycerol; PI, phosphatidylinositol; PL, phospholipids; TAG, triacylglycerol. Chloroplast and endoplasmic reticulum glycerolipid pathways are indicated by the transfer of fatty acids from acyl-ACP and acyl-CoA, respectively. Key metabolism genes are indicated by red italics: DGAT, DAG acyltransferase; GK, glycerol kinase; GPAT, G3P acyltransferase; GPD, G3P dehydrogenase; GPP, G3P phosphatase; LPAAT, lyso-PA acyltransferase; PAP, PA phosphatase; PDAT, phospholipid:DAG acyltransferase. In (B), the early, middle, and late stages of flower bud differentiation are respectively denoted as FE, FM, and FL (‘Nagafu No. 2’) and QE, QM, and QL (‘Qinguan’). Data are presented as the mean ± standard error, *n* = 3. *p < 0.05; **p < 0.01; ***p < 0.001; ns, non-significant (*p* > 0.05)

To confirm the RNA-seq results, we completed a qRT-PCR assay to examine the expression levels of carbohydrate-, hormone-, and flowering-related genes in buds during different developmental stages (ES, MS, and LS) in both ‘Qinguan’ and ‘Nagafu No. 2’ buds (Figs. 7, 10, and 12). The linear relationship (*R*^*2*^ = 0.7962, *p* < 0.01) between the qRT-PCR results and the RNA-seq data for related genes in buds is shown in Additional file 1: Figure S10.

### Predominance of carbohydrate, fatty acid, and lipid pathways among specialized metabolic pathways differentially expressed between ‘Qinguan’ and ‘Nagafu No. 2’ buds

The functions of genes differentially expressed between ‘Qinguan’ and ‘Nagafu No. 2’ buds during floral induction were centred around carbohydrate and lipid complex metabolic networks (Fig. 7 and Additional file 2). For example, *HXK2*, *PGI* (2), *PFK3*, *PFK7*, and *FBA2* genes, which convert glucose to glycerol-3-phosphate (G-3-P) in carbohydrate metabolism, had significantly higher transcript accumulations in ‘Qinguan’ than in ‘Nagafu No. 2’ buds (Fig. 7). The expression levels of three sucrose synthesis-related genes, *SPS1* and *SPS3* genes in the ES and the *SUS3* gene in the MS, were significantly higher in ‘Qinguan’ buds than in ‘Nagafu No. 2’ buds (Fig. 7). The *FRK* gene, which is important for fructose-6-phosphate biosynthesis, had significantly higher expression levels in ‘Qinguan’ buds than in ‘Nagafu No. 2’ buds (Fig. 7). Three *TPI* genes had significantly higher expression levels in ‘Qinguan’ than in ‘Nagafu No. 2’ from the ES to the LS (Fig. 3). Six key *TPS* genes (three *TPS7* genes and three *TPS9* genes) involved in T6P synthesis, two key *TPP2* genes involved in trehalose synthesis, and *TRE1* had significantly lower expression levels in ‘Qinguan’ buds than in ‘Nagafu No. 2’ buds (Fig. 7). Genes related to starch biosynthesis, including *SS3*, *SS4*, *SBE2*, and *BE1*, had significantly decreased expression levels in ‘Qinguan’ buds compared with ‘Nagafu No. 2’ buds (Fig. 7), with three starch degradation genes (*AMY2*, *BMYB*, and *BMY3*) exhibiting the opposite pattern (Fig. 7).

The *PK*, *ENO1*, and *ENO2* genes, which are associated with the biosynthesis of pyruvate, a precursor of acetyl-CoA in the Calvin cycle, have the key function of linking carbohydrate, nitrogen, and lipid pathways. These genes were significantly more highly expressed in ‘Qinguan’ buds than in ‘Nagafu No. 2’ buds (Fig. 7).

Two key genes associated with malonyl-CoA biosynthesis (*PDH* and *ACC1*) and the *MAT3* gene involved in maloyl-ACP biosynthesis were significantly more highly expressed in ‘Qinguan’ buds than in ‘Nagafu No. 2’ buds (Fig. 7). Meanwhile, *KAS1*, *KASIII* (2), *KAS2* (4), and *ENR1* (2), which are involved in fatty acid biosynthesis, were significantly more highly expressed in ‘Qinguan’ buds than in ‘Nagafu No. 2’ buds (Fig. 7). The same was true of *LACS2*, *LACS9* (2), *ACX1* (3), *ACX2* (2), and *KAT2* (2), which are involved in the formation of acyl-CoA pools from fatty acid, an important substance in oil synthesis (Fig. 7). Transcriptional levels of TAG assembly pathway genes, including *GPAT3*, *GPAT5*, *GPAT9* (2), *LPAT2* (2), *PAP2* (2), and *PDAT*, were higher in ‘Qinguan’ than in ‘Nagafu No. 2’ (Fig. 7), with the opposite trend observed for the *DGAT* gene (Fig. 7).

The following three genes related to lipid metabolic pathways had significantly higher expression levels in ‘Qinguan’ buds than in ‘Nagafu No. 2’ buds: *PIS1* (2) associated with phosphatidylinositol (PI) formation and *PSD1* associated with phosphatidylglycerol (PG) formation (Fig. 7). The *MGD1* and *MGD2* genes involved in MGDG formation and a *DGD1* gene contributing to DGDG synthesis were more highly expressed in ‘Qinguan’ buds than in ‘Nagafu No. 2’ buds (Fig. 7). Moreover, three genes, *SQD1* (2) and *SQD2*, respectively involved in the formation of SQDG and ASQD following diacylglycerol (DAG) degradation, were significantly more highly expressed in ‘Qinguan’ than in ‘Nagafu No. 2’ (Fig. 7).

### Differential expression of hormone metabolism and signalling pathways in ‘Qinguan’ buds compared with ‘Nagafu No. 2’ buds during floral induction

**Fig. 8:**
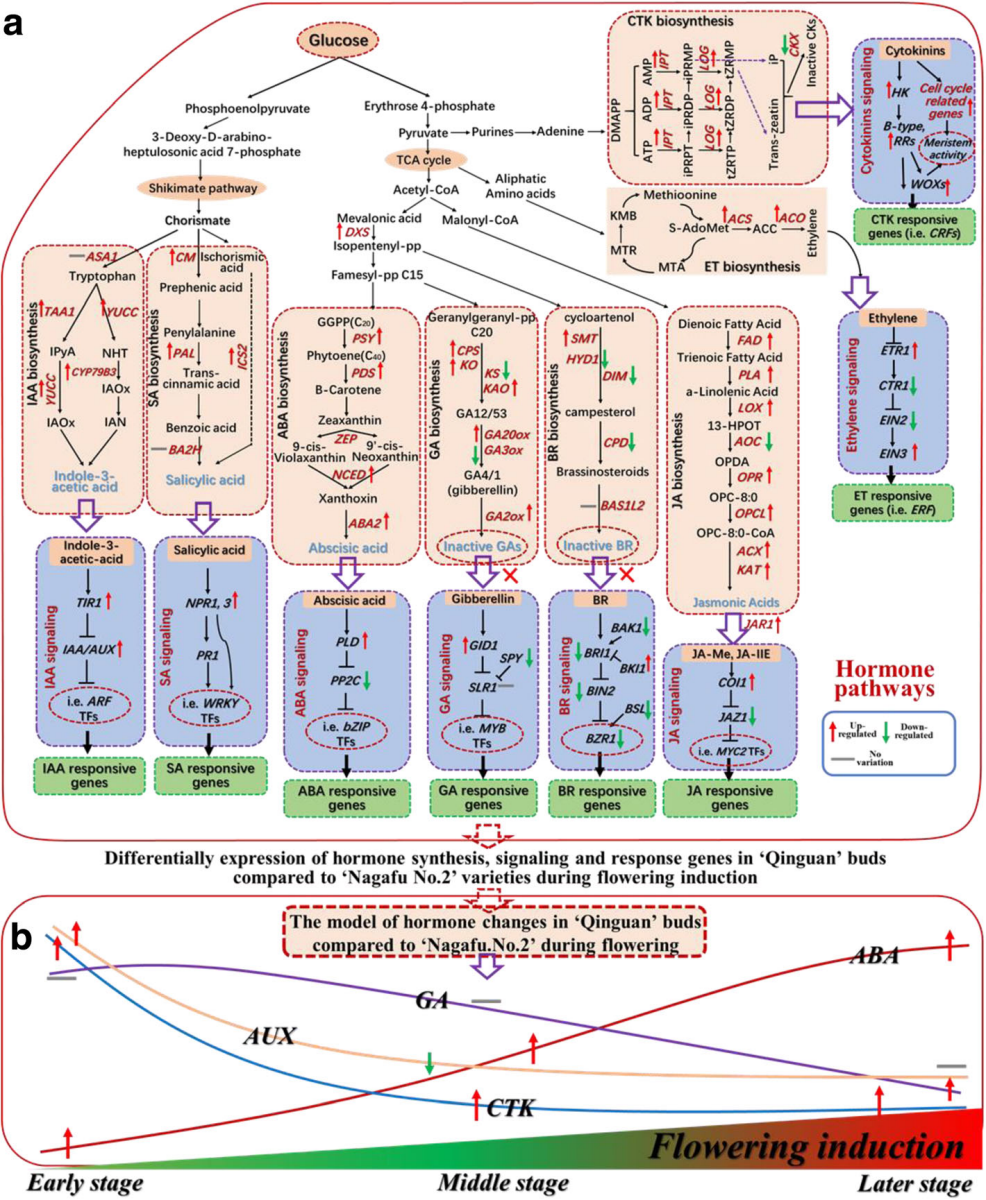
Differential expression of genes involved in hormone synthesis, signalling, and response pathways in ‘Qinguan’ and ‘Nagafu No. 2’ buds as reconstructed from transcriptomic evidence. **a** Model of the differential expression of genes involved in hormone synthesis, signalling, and response pathways in profusely flowering ‘Qinguan’ and weakly flowering ‘Nagafu No. 2’. Expression trends are represented by arrows. **b** Model of hormone changes in ‘Qinguan’ and ‘Nagafu No. 2’ buds during floral induction

**Fig. 9:**
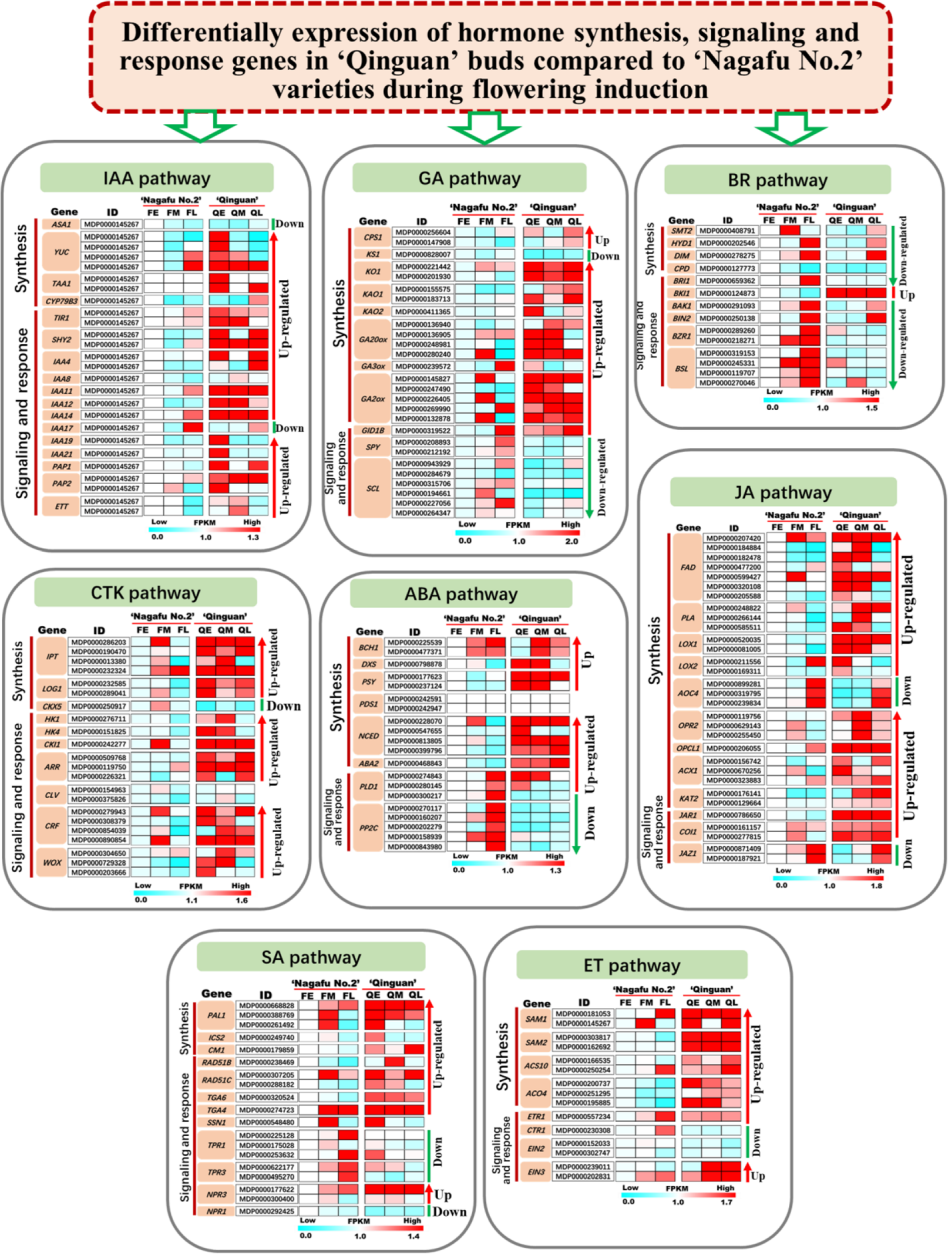
Results of a cluster analysis of hormone synthesis-, signalling-, and response-related genes linked to the model in Fig. 11. Early, middle, and late stages of flower bud differentiation are respectively denoted as FE, FM, and FL in ‘Nagafu No. 2’ and QE, QM, and QL in ‘Qinguan’

**Fig. 10:**
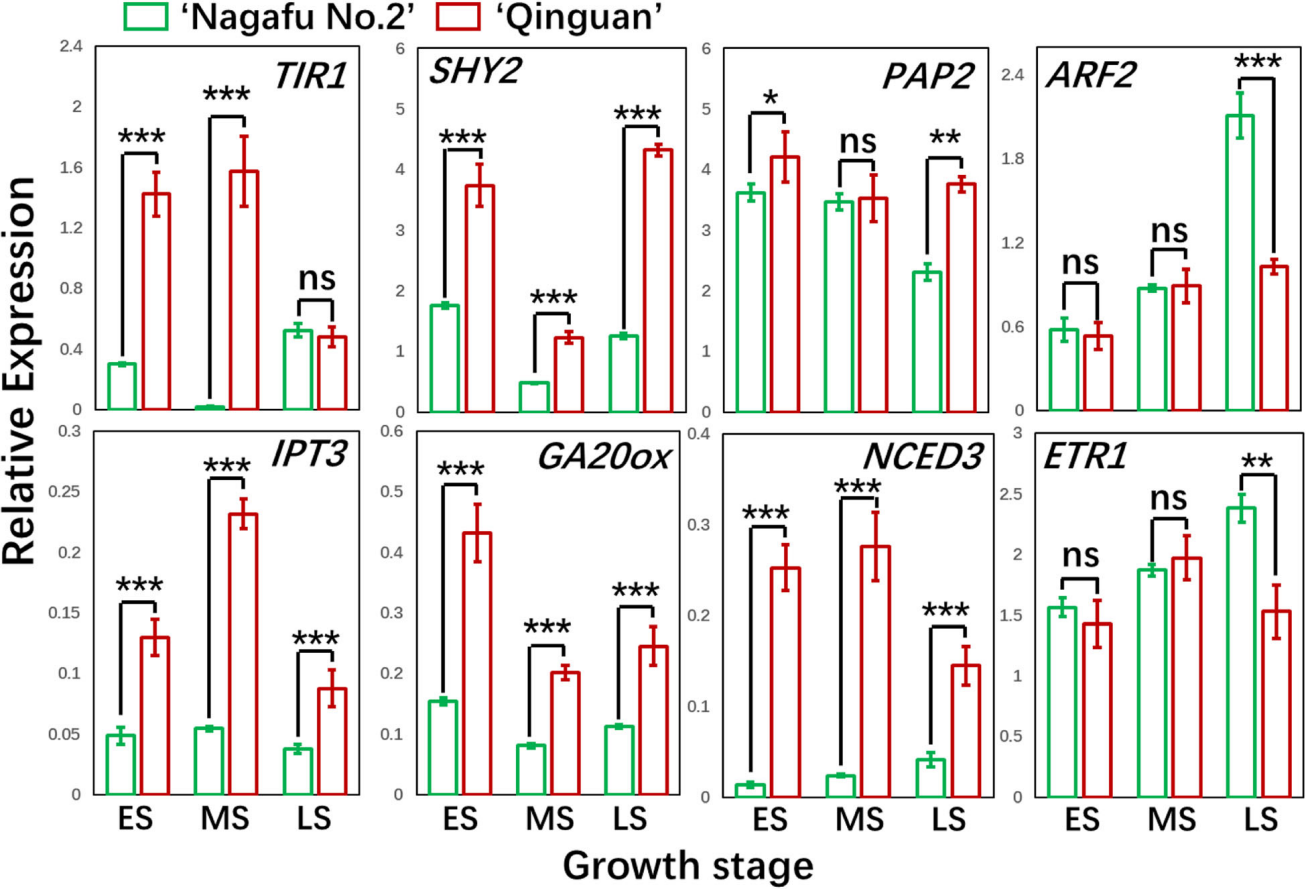
Identification by quantitative real-time PCR of differentially expressed genes associated with hormone signalling pathways in ‘Qinguan’ and ‘Nagafu No. 2’ buds during floral induction. ES, MS, and LS correspond to the early, middle, and late stages of flower bud differentiation, respectively. Data are presented as the mean ± standard error, *n* = 3. *p < 0.05; **p < 0.01; ***p < 0.001; ns, non-significant (*p* > 0.05)

Our comparative transcriptome and cluster analyses of DEG expression profiles of ‘Qinguan’ and ‘Nagafu No. 2’ buds revealed the existence of complex regulatory networks associated with multiple hormone synthesis, dynamics, and signalling pathways (Figs. 8, 9, 10 and Additional file 2). Genes downstream of the shikimate pathway, namely, indole-3-acetic acid (IAA) biosynthesis genes, such as *YUCC* (4) and *TAA1* (2) during the ES and *CYP79B3* during the LS, and SA biosynthesis genes, including *PAL1* (3) and *ICS2* during the ES and *CM1* during the ES and LS, were significantly more highly expressed in ‘Qinguan’ buds than in ‘Nagafu No. 2’ buds (Figs. 8, 9, 10). Additionally, the expression levels of *TIR1* (2) during the ES and MS as well as AUX/IAA transcriptional regulator family genes [i.e., *IAA4* (2)*, 8, 11, 12, 14, 19, 21*, *SHY2* (2), *PAP1,* and *PAP2* (*2*)] associated with IAA signalling were also significantly up-regulated in ‘Qinguan’ relative to ‘Nagafu No. 2’ (Figs. 8, 9, 10). *SMALL AUXIN UP RNAs* (*SAURs*), which constitute the largest family of early auxin response genes, were differentially expressed between ‘Qinguan’ and ‘Nagafu No. 2’ buds. Specifically, 12 *SAURs* (e.g., *M286931, M186167, M285050*, and *M668689*) were more highly expressed in ‘Qinguan’ than in ‘Nagafu No. 2’ (Additional file 1: Figure S7). Additionally, the expression levels of genes such as *TGA4* and *TGA6*, which are involved in SA signalling and response, were significantly up-regulated in ‘Qinguan’ buds relative to ‘Nagafu No. 2’ buds (Figs. 8 and 9).

Among genes functioning downstream of the tricarboxylic acid (TCA) cycle pathway, ABA biosynthesis genes [i.e., *DXS PSY* (2), *NCED* (4), and *ABA2* from the ES to the LS] and JA biosynthesis genes [e.g., *FAD* (7) during the ES and MS; *PLA* (3), *LOX1* (2), *OPCL1, KAT2* (2), and *ACX1* (3) from the ES to the LS; and *OPR2* (3) during the MS and LS)] were significantly more highly expressed in ‘Qinguan’ buds than in ‘Nagafu No. 2’ buds (Figs. 8 and 9). The expression levels of two *PLD1* genes involved in ABA signalling (*M274843* and *M280145*) were significantly up-regulated during the ES in ‘Qinguan’ buds relative to ‘Nagafu No. 2’ buds (Figs. 8 and 9). The opposite pattern was observed for *PP2C* (5) during the MS and LS (Figs. 8 and 9). Moreover, *JAR1* and *COI1* (2), which are involved in JA signalling, were significantly more highly expressed in the ‘Qinguan’ buds than in the ‘Nagafu No. 2’ buds from the ES to the LS (Figs. 8 and 9), with the opposite pattern observed for *JAZ1* (2) during the ES and MS (Figs. 8 and 9).

Among genes in the GA biosynthesis pathway, the following were significantly more highly expressed in ‘Qinguan’ buds than in ‘Nagafu No. 2’ buds: *CPS1* (2) during the MS and LS, *KAO1* (2) and *KO1* (2) from the ES to the LS, *KAO2* during the ES, and *GA20ox* (4) during the ES and MS (Figs. 8, 9, 10). In contrast, *GA3ox* was more highly expressed in ‘Nagafu No. 2’ than in ‘Qinguan’ during the LS (Figs. 8). Meanwhile, five *GA2ox* genes (*M145827*, *M247490, M226405, M269990,* and *M132878*)*,* which are important for the synthesis of inactive GAs, were more highly expressed in ‘Qinguan’ than in ‘Nagafu No. 2’ (Figs. 8 and 9). Furthermore, genes related to GA signalling and response pathways, namely *SCARECROW-like* family genes (e.g., *M943929, M227056*, and *M264347*) from the ES to the LS and two *SPY* genes (*M208893* and *M212192*) during the MS and LS, exhibited the opposite trend (Figs. 8 and 9). Regarding the brassinosteroid (BR)-specific biosynthesis pathway, *SMT2*, *DIM*, and *CPD* during the ES and MS and *HYD1* from the ES to the LS had significantly lower expression levels in ‘Qinguan’ buds than in ‘Nagafu No. 2’ buds (Figs. 8 and 9). The expression levels of several important BR signal transduction genes, including *BRI1* during the LS, *BAK1* from the ES to the LS, and *BZR1* (2) and *BSL* (4) during the MS and LS, were significantly down-regulated in the ‘Qinguan’ buds compared with the ‘Nagafu No. 2’ buds (Figs. 8 and 9), while the opposite pattern was observed for *BKI1* from the ES to the LS (Figs. 8 and 9).

The expression levels of ethylene biosynthesis pathway-related genes, including *SAM1* (2) during the ES and LS, *SAM2* (2), *ACS10* (2), and *ACO4* (3) from the ES to the LS, and ET signalling genes, including *ETR1*, during the ES and MS, and *EIN3* (2), were significantly up-regulated in ‘Qinguan’ buds relative to ‘Nagafu No. 2’ buds (Figs. 8, 9, 10). In contrast, the *CTR1* expression level exhibited the opposite trend (Figs. 8 and 9). Similarly, *IPT* (*M286203*, *M190470*, *M013380*, and *M232324*) and *LOG1* (*M232585* and *M289041*) genes, which are associated with CTK biosynthesis, were significantly more highly expressed in ‘Qinguan’ buds than in ‘Nagafu No. 2’ buds from the ES to the LS (Figs. 8 and 9), with the opposite pattern observed for the inactive CTK biosynthesis gene *CKX5* (Figs. 8 and 9). Genes involved in CTK signalling, namely *HK1* and *HK4* during the ES and MS and *CKI1* during the ES and LS, were significantly more highly expressed in ‘Qinguan’ buds than in ‘Nagafu No. 2’ buds (Figs. 8 and 9). A similar result was observed for CTK response genes such as *CRF* (4) and *WOX* (3) from the ES to the LS (Figs. 8 and 9). Additionally, approximately four clusters of DEGs associated with cell division, cell differentiation, and the cell cycle (i.e. *CYC* and *CDK* genes) were identified (Additional file 1: Figure S8). The expression levels of genes from the following two clusters were significantly up-regulated in the ‘Qinguan’ buds compared with the ‘Nagafu No. 2’ buds: a-cluster containing nine genes (e.g., *CYCD6:1, CYCA3;1, CYCD3:1*, and *CYCD3:2*) from the ES to the LS and c-cluster comprising 20 genes (e.g., *CDKB1:2*, *CDKB2:2*, *CDKB2:4*, *CYCD1:1*, and *CYCD2:1*) during the ES and LS (Additional file 1: Figure S8). In contrast, the expression levels of genes in the b-cluster (e.g., *CYCH:1*, *CYCD1:3*, and *CYCD5:1*) were significantly up-regulated in ‘Nagafu No. 2’ (Additional file 1: Figure S8).

### Responses of TFs differentially expressed between ‘Qinguan’ and ‘Nagafu No. 2’ buds during floral induction

On the basis of our transcriptome data, we identified 500 TFs belonging to 45 families in which each TF family member was differentially expressed between ‘Qinguan’ and ‘Nagafu No. 2’ buds during the floral induction process (Additional file 1: Figure S9 and Additional file 2). Significantly lower expression levels were observed in ‘Qinguan’ relative to ‘Nagafu No. 2’ from the ES to the LS for several TF genes, most of which were from the *bHLH* (50), *ERF* (43), and *WRKY* (37) TF families (Additional file 1: Figure S9), although there were some from the *CPP* (1), whirly (1), *VOZ* (2), and *DBB* (2) TF families (Additional file 1: Figure S9). Some *WRKY* family genes involved in stress responses and floral development, such as *M268364*, *M228304*, *M175240, M253189,* and *M496268*, were significantly more highly expressed in ‘Qinguan’ than in ‘Nagafu No. 2’ during the MS and LS (Additional file 1: Figure S9). Similarly, the expression levels of key *bZIP* family genes associated with the ABA response (*M320524*, *M169473, M891899,* and *M863909*) as well as some NAC family genes (*M121265*, *M690168*, *M239596, M138340,* and *M868556*) were up-regulated in ‘Qinguan’ buds compared with ‘Nagafu No. 2’ buds (Additional file 1: Figure S9). In contrast, the expression levels of *AP2* family genes (*M296716*, *M181606*, *M130802*, and *M161347*) associated with flower and fruit development and phase transitions were down-regulated in ‘Qinguan’ buds compared with ‘Nagafu No. 2’ buds during the ES (Additional file 1: Figure S9). Additionally, several *IDD* family members (*M373134, M233477*, *M171492,* and *M122321*) associated with C2H2 TFs involved in the regulation of flowering in sugar pathways were more highly expressed in ‘Qinguan’ buds than in ‘Nagafu No. 2’ buds (Additional file 1: Figure S9). These differentially expressed TF genes related to multiple regulatory pathways may be useful for regulating floral induction in ‘Qinguan’ and ‘Nagafu No. 2’ apple trees.

### Differential expression of flowering pathway genes between ‘Qinguan’ and ‘Nagafu No. 2’ buds during floral induction

**Fig. 11:**
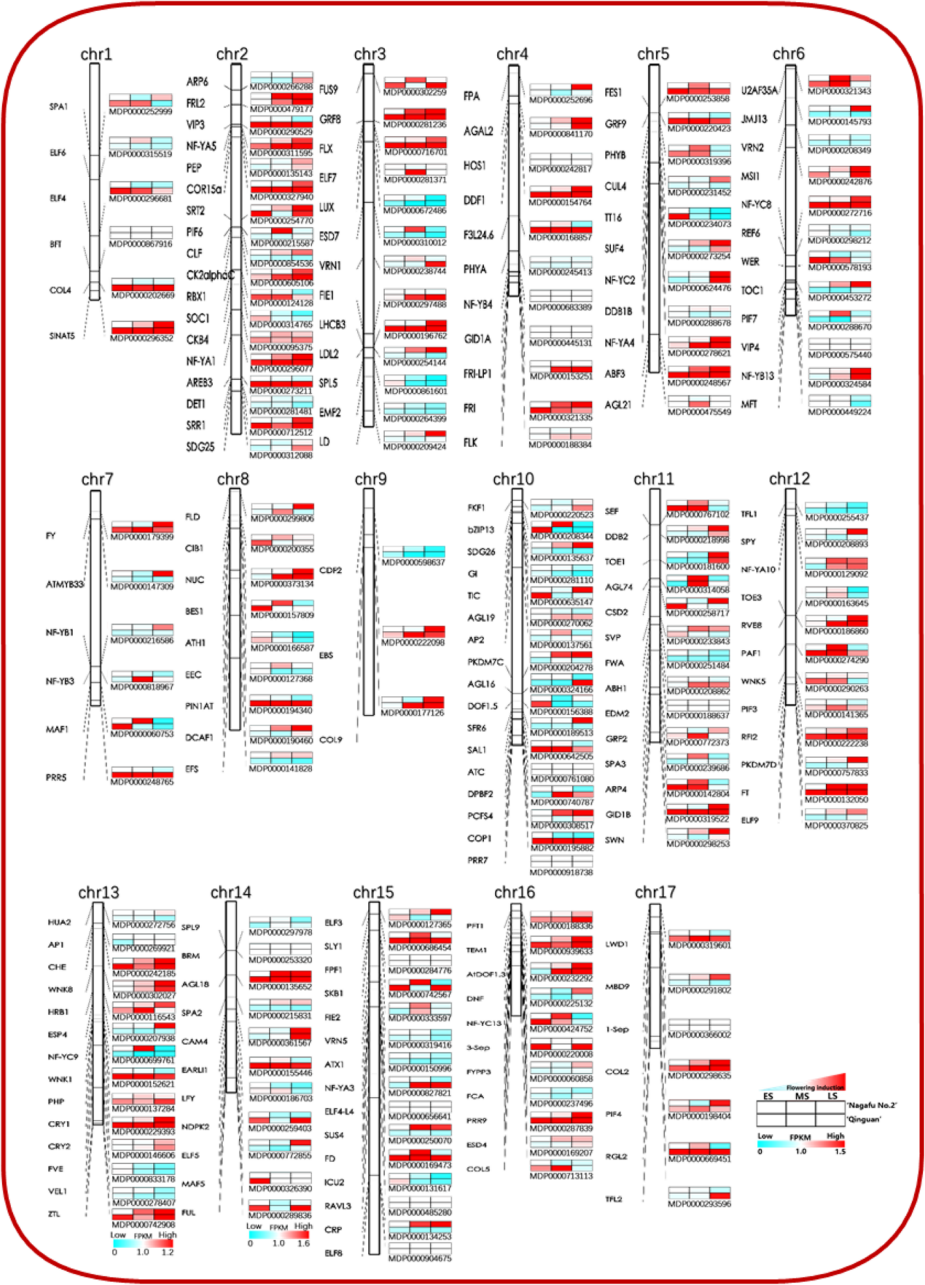
Results of a cluster analysis of differentially expressed flowering-related genes located on different chromosomes in ‘Qinguan’ and ‘Nagafu No. 2’ buds during floral induction. Early, middle, and late stages of flower bud differentiation are respectively denoted as FE, FM, and FL in ‘Nagafu No. 2’ and QE, QM, and QL in ‘Qinguan’. See Additional file 5 for more information regarding expression profiles and full annotations

**Fig. 12:**
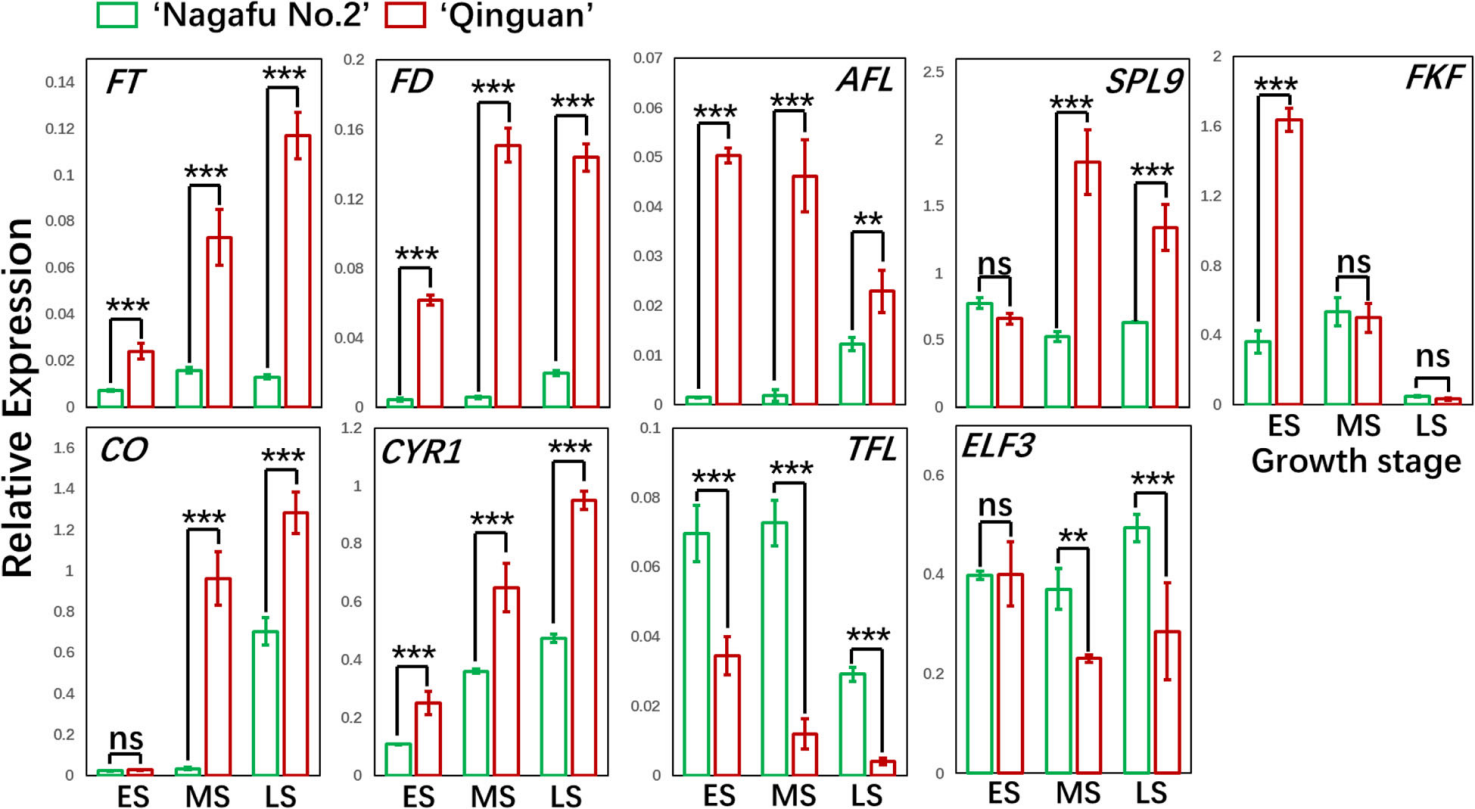
Identification by quantitative real-time PCR of differentially expressed flowering-related genes in ‘Qinguan’ and ‘Nagafu No. 2’ buds during floral induction. ES, MS, and LS correspond to the early, middle, and late stages of flower bud differentiation, respectively. Data are presented as the mean ± standard error, *n* = 3. *p < 0.05; **p < 0.01; ***p < 0.001; ns, non-significant (*p* > 0.05)

Genetic linkage maps involving 190 flowering genes were constructed to clarify the association between ‘Nagafu No. 2’ and ‘Qinguan’ (Figs. 11 and 12 and Additional file 2). The expression profiles of these flowering genes on the 17 apple chromosomes were significantly different between ‘Nagafu No. 2’ and ‘Qinguan’ (Fig. 11). Several important genes involved in floral development were significantly more highly expressed in ‘Qinguan’ buds than in ‘Nagafu No. 2’ buds. These included the floral meristem identity control protein gene *LEAFY* (*M186703*) on chromosome 14 (from the ES to the LS), the flowering time regulatory protein genes PFT1 (*M188336*) on chromosome 16 and *FT* (*M132050*) on chromosome 12 (from the ES to the LS), the basic-leucine zipper TF family protein gene *FD* (*M169473*) on chromosome 15 (from the ES to the LS), and the MADS-box genes *FUL* (*M289836*) on chromosome 14 (during the ES and LS) and *SOC1* (*M314765*) from ES to LS on chromosome 2 (Figs. 11 and 12). Other genes whose expression levels were similarly significantly higher in ‘Qinguan’ than in ‘Nagafu No. 2’ included several *SQUAMOSA PROMOTER BINDING PROTEIN-LIKE* (*SPL*) genes [*SPL5* (*M861601*) during LS on chromosome 3 and *SPL9* (*M29978*) during LS on chromosome 14, several circadian rhythm-related genes [*CRY1* (*M229393*) on chromosome 13 (from the ES to the LS), *PRR5* (*M248765*) on chromosome 7 (from the ES to the LS), and *PIF3* (*M141365*) on chromosome 12 (from the ES to the LS)], and *CO* genes [*M713113* on chromosome 16 (during the ES and MS) and *M177126* on chromosome 3 (during the MS)] associated with photoperiodism and flowering (Figs. 11 and 12). In contrast, the following genes were expressed at significantly lower levels in ‘Qinguan’ buds than in ‘Nagafu No. 2’ buds: PEBP family gene *TFL1* (*M255437*) on chromosome 12 (during the ES and MS), MADS-box TF gene *SVP* (*M233843*) on chromosome 11 (during the MS and LS), the cycling DOF factor 2 gene *CDF2* (*M598637*) during MS and LS on chromosome 9, *ELF3* (*M127365*) on chromosome 15 (during the MS and LS), and *AP2* (*M137561*) on chromosome 10 (during the MS and LS) (Figs. 11 and 12).

## Discussion

### Differences in agronomic and floral-associated traits between profusely flowering ‘Qinguan’ and weakly flowering ‘Nagafu No. 2’

‘Qinguan’, an apple variety developed in China in 1960, exhibits profuse flowering, high yields, and strong stress and disease resistance [4, 25], whereas the apple variety ‘Nagafu No. 2’ forms flower buds with difficulty and has the disadvantages of exhibiting the alternate bearing phenotype and vigorous vegetative growth [26, 27]. Consistent with these properties, we observed that flowering and bud break rates of ‘Qinguan’ trees were significantly higher than those of ‘Nagafu No. 2’ trees from 2013 to 2015 (Fig. 2). These results suggest the existence of large differences in floral-related traits between the two apple varieties. When we compared the bud growth of ‘Qinguan’ and ‘Nagafu No. 2’, we found that bud width and fresh weight in the middle and late stages of flower bud physiological differentiation were significantly higher in ‘Qinguan’ (Fig. 1), thereby contributing to its superior flowering characteristics. Other researchers have reported similar findings [1, 27]. Previous studies have also revealed that the relatively weak vegetative growth of woody fruit trees is due to early cessation of vegetative shoot growth [1]. Additionally, shoot bending can significantly promote flower bud formation by inhibiting the vegetative growth of the tree and reducing the proportion of long shoots [19]. A similar conclusion can be inferred from our results (Fig. 2), which suggest that the high proportion of short shoots and the weak vegetative growth of ‘Qinguan’ trees contribute to the higher number of flower buds in this variety.

### Significant differences in bud sugar and hormone levels between profusely flowering ‘Qinguan’ and weakly flowering ‘Nagafu No. 2’ apple varieties during floral induction

As the main source of energy, sugars participate in the plant flowering process [28]. Carbohydrate metabolism, which involves the synthesis, catabolism, and mutual transformation of starch and sugars, such as sucrose, has an important role in plant growth, floral induction, and other processes [9, 29]. In our study, significant differences were found in the contents of various sugars during floral induction between the ‘Qinguan’ and ‘Nagafu No. 2’ buds, which contributed to the contrasting levels of bud growth and flower bud formation observed between the two varieties (Fig. 3). A similar study has suggested that changes in sugar composition induced by shoot bending in apple buds can contribute to accelerated flower bud formation [30].

Phytohormones play key roles in the complex regulation of floral transitions [31]. We detected significant differences in plant hormones (i.e. CTK, ABA, GA, and auxin) between the buds of profusely flowering ‘Qinguan’ and weakly flowering ‘Nagafu No. 2’ (Fig. 4). A previous study confirmed that CTK is mainly involved in the initiation of flower bud formation in plants [6], which is consistent with the results of our study, where CTK levels were highest in the early stages—decreasing from the ES to the LS in both varieties (but remaining significantly higher in ‘Qinguan’ than in ‘Nagafu No. 2’) (Fig. 4). This suggests that CTK plays a positive role in flower bud formation. Similarly, ABA levels were significantly higher in ‘Qinguan’ buds than in ‘Nagafu No. 2’ buds throughout all three bud differentiation stages. An earlier investigation revealed that ABA contents increase during flower bud differentiation, especially during flower organogenesis [32]. Moreover, ABA helps regulate seed development and floral and phase transitions in response to environmental stresses [18]. In contrast, the IAA content of ‘Qinguan’ buds was significantly higher than that of ‘Nagafu No. 2’ buds only during the ES (Fig. 4). Previous research has indicated that IAA affects bud growth and development to help regulate floral induction [33]. Similar to the findings of other studies [21, 22], our data indicate that GA has a negative regulatory role in floral induction in woody fruit trees (Fig. 4).

### Differentially expressed genes involved in carbohydrate and lipid pathways contributing to differences in flower bud formation between profusely flowering ‘Qinguan’ and weakly flowering ‘Nagafu No. 2’

An analysis of the global transcriptome data revealed that the majority of DEGs were related to complex regulatory networks involving carbohydrate, nitrogen, and lipid pathways, with pyruvate as the transit station (Fig. 7 and Additional file 2). The two diverging paths at the end of the pathway responsible for the conversion of fructose to G-3-P in the whole gene regulatory network of carbohydrate metabolism may explain the higher *HXK*, *PGI*, *FRK*, and *FBA* expression levels in ‘Qinguan’ buds than in ‘Nagafu No. 2’ buds (Fig. 7). The first path, responsible for the down-regulated expression of starch synthesis genes (i.e., *SS3*, *SS4*, *SBE2*, and *BE1*) in ‘Qinguan’ buds, involves the synthesis of starch using T-6-P as an intermediate. Earlier studies concluded that T-6-P can serve as a proxy for carbohydrate status in plants and affect flowering by regulating *FT* expression [9, 18]. Additionally, T-6-P reportedly acts as a centre for regulators involved in carbohydrate metabolism, such as sucrose synthesis and starch metabolism [34]. Additionally, the greater abundance of sucrose and the higher expression levels of sucrose synthesis genes (i.e., *SPS1*, *SPS3*, and *SUS3*) in ‘Qinguan’ buds than in ‘Nagafu No. 2’ buds during the initial floral induction stage may contribute to the enhanced flower bud formation in ‘Qinguan’ (Fig. 7). A similar study has revealed that the application of a 1% (*w*/*v*) sucrose solution promotes flowering [35], while the up-regulated expression of sucrose synthase genes (*SUS1* and *SUS4*) in 35S:IDD8 plants enhances photoperiodic flowering [11]. The second possible path involves the conversion of G-3-P to pyruvate in the sugar pathway (Fig. 7), followed by lipid and nitrogen metabolic pathways (Fig. 7). This path involves the up-regulated expression of *PGK*, *ENO*, and *PK* in ‘Qinguan’ buds, which suggests that differences in the expression levels of these carbohydrate-related genes between the profusely flowering ‘Qinguan’ and the weakly flowering ‘Nagafu No. 2’ contribute to the contrasting levels of flower bud formation in these two apple varieties.

Genes such as *PHD*, *ACC1*, *KASI*, and *KASIII* are expressed downstream of pyruvate in the fatty acid synthesis pathway and are involved in the synthesis and metabolism of certain compounds (i.e., acetyl-CoA, malonyl-CoA, and acyl-ACP). These genes were more highly expressed in ‘Qinguan’ buds than in ‘Nagafu No. 2’ buds (Fig. 7). Compared with their fate in ‘Nagafu No. 2’ buds, energy and photosynthetic products are more likely to be converted into fatty acids via acyl-ACP and then channelled into lipid pathways in ‘Qinguan’ (rather than direct starch formation through central carbon pathways). Additionally, lipids reportedly influence signalling pathways that control plant reproductive development [12, 36].

The greater expression of the key genes *LACS2* and *LACS9* in ‘Qinguan’ buds than in ‘Nagafu No. 2’ buds leads to the conversion of more fatty acids into acyl-CoA in the cytoplasm/ER and, together with G-3-P from the TCA cycle (Fig. 7), entry into complex lipid synthesis and dynamics pathways [36]. This situation suggests that the decomposition and synthesis of these metabolites, which include phosphatidic acid (PA), phosphatidyl-ethanolamine (PE), phosphatidylcholine (PC), phosphatidylglycerol (PG), phosphatidylinositol (PI), and triacylglycerol (TAG), occur more in ‘Qinguan’ buds than in ‘Nagafu No. 2’ buds, thereby contributing to the higher flowering rates in ‘Qinguan’ (Fig. 7). Research has shown that FT binds to diurnally changing molecular PC species, mainly 18:1-PC, in the shoot apex to promote flowering [12]. Overexpression of *FATTY ACID DESATURASE3* (*FAD3*) to yield a higher proportion of 18:3-PC relative to 18:1-PC delays flowering [12, 13]. Additionally, genes involved in lipid deposition and oil (TAG) formation (i.e. *PDAT* and *PAP2*) were significantly more highly expressed in ‘Qinguan’ buds than in ‘Nagafu No. 2’ buds (Fig. 7). This result is similar to the findings of an earlier study on *Brassica napus*, in which Wrinkled1, a central regulator of oil synthesis, was observed to accelerate plant flowering by regulating lipid homeostasis between oil accumulation and lipid anabolism [13]. Wrinkled1 also accelerates flowering by enhancing *FT* expression and increasing PC levels [12, 13].

### Differentially expressed genes involved in hormone metabolism and signalling pathways contributing to differences in flower bud formation between profusely flowering ‘Qinguan’ and weakly flowering ‘Nagafu No. 2’

Towards the elucidation of the regulatory mechanism of hormones during floral induction, we comprehensively analysed the genes involved in eight plant hormone metabolic, signalling, and response pathways that were differentially expressed between the profusely flowering ‘Qinguan’ and weakly flowering ‘Nagafu No. 2’ (Fig. 8). These DEGs included genes such as *YUCC* and *TAA1*, which are responsible for the synthesis of IAA and SA via the shikimate pathway [37]. The expression levels of the key IAA biosynthesis genes were initially up-regulated in ‘Qinguan’ buds (Figs. 8, 9, 10), which indicates that IAA plays a key role in bud growth and the initiation of floral induction. Relatively high IAA levels in buds can stimulate bud growth and positively affect the initiation of floral induction [33]. Moreover, SA reportedly plays a positive role in flowering, mainly through its involvement in stress responses [38]. This hormone also regulates flowering by affecting the expression of key floral genes (i.e., *FLC* and *FT*) [16, 24] as well as SA signalling and response genes, including *NPR1* [16]. A similar inference can be made from our data (Fig. 8), suggesting the superior floral characteristics of ‘Qinguan’ are closely related to the stronger resistance conferred by genes in the SA signalling and flowering pathways.

The role of CTK in flowering has been previously studied [6, 14]. The application of exogenous CTK can significantly increase the apple flowering rate [19]. We observed significantly higher CTK levels and up-regulated expression levels of CTK biosynthesis genes and some cell cycle-related genes (*CYCA3;1* and *CYCP1;1*) in profusely flowering ‘Qinguan’ buds during floral induction (Additional file 1: Figure S8). Thus, CTK appears to affect flower bud formation in this variety. Other studies have shown that CTK regulates floral induction by up-regulating the expression of floral-related genes such as *FT* [39] and *SOC1* [6, 20]. In our study, the expression levels of floral-related genes, such as *FT*, *FD*, and *SOC1*, were up-regulated in ‘Qinguan’ to levels similar to those of CTK (Figs. 11 and 12). Additionally, the proteins encoded by B-type *ARR* genes (CTK-responsive genes) combine with SPLs to activate the expression of *SOC1* and *AGL24*, which are associated with CTK signalling [40]. We observed that the B-type *ARR* genes were more highly expressed in ‘Qinguan’ buds than in ‘Nagafu No. 2’ buds (Figs. 8 and 9). Moreover, the expression levels of genes involved in ethylene synthesis and signalling (*SAM1*, *ACS10*, and *ETR1*) were up-regulated in ‘Qinguan’ buds (Figs. 8 and 10). Ethylene has a key role in the regulation of floral induction [41].

Downstream of the TCA cycle pathway, ABA and JA, which are involved in stress responses, have positive roles in the regulation of floral induction and formation [16]. The main function of ABA signalling in flowering involves the regulation of circadian rhythms and the expression of photoperiod-related genes such as *EDL3*, *CO*, and *GI* [15, 42]. Additionally, GI can also induce flowering by increasing *FT* and *TFS* expression levels in response to ABA signalling [43, 44]. Furthermore, ABA-linked sugar signalling influences the regulation of vegetative development, flowering time, and stress responses in plants [18]. The expression levels of genes related to ABA biosynthesis and flowering (*FT*, *SOC1*, and *CO*) were up-regulated in profusely flowering ‘Qinguan’ (Fig. 12), implying that ABA is a central factor involved in multiple pathways (i.e., sugar, photoperiodic, and circadian rhythm pathways) that help regulate floral induction. Another plant hormone, JA, induces flowering by regulating responses to biotic and abiotic stresses along with COI1 and JAZ from the photoperiodic pathways [16]. Consistent with this finding, the higher flowering rate of ‘Qinguan’ trees relative to ‘Nagafu No. 2’ trees was closely coupled to the up-regulated expression of genes involved in JA synthesis (i.e., *FAD*, *PLA*, *LOX*, and *KAT*) (Figs. 8 and 9).

In contrast to the above-mentioned hormones, exogenous GA inhibits flowering in woody fruit tree species (e.g., mango and apple trees) by repressing *FT* expression [45, 46]. We similarly observed that GA levels and the expression of *GA3ox*, a key GA synthesis gene, were significantly lower in ‘Qinguan’ buds than in ‘Nagafu No. 2’ buds (Figs. 8 and 9). Another plant hormone, BR, which is mainly involved in plant growth and development, has the opposite effect on flowering and is associated with the regulation of *FLC, LD*, and *FCA* expression in the autonomous pathway [47]. Our data suggest that the down-regulated expression of genes involved in BR synthesis (*SMT2*, *DIM*, and *CPD*) in ‘Qinguan’ trees contributes to the high flowering rate of this apple variety (Figs. 8 and 9). Thus, the molecular regulatory mechanisms underlying floral induction may include complex regulatory processes involving multiple plant hormones.

### Comparative global analysis of TFs potentially involved in the regulation of flower bud formation in profusely flowering ‘Qinguan’ and weakly flowering ‘Nagafu No. 2’

The plant life cycle involves many biological processes, such as cell division and differentiation, embryonic development, seed germination, reproductive growth, and flowering, that are regulated by complex transcriptional networks [48]. Approximately 500 differentially expressed TFs belonging to 45 families were identified between ‘Qinguan’ and ‘Nagafu No. 2’ buds during the floral induction process (Additional file 1: Figure S9 and Additional file 2). Some of these differentially expressed TFs are related to hormone responses, such as bZIPs, ERFs, ARFs, and MYBs [49–51]. Other identified TFs (WRKYs and NACs) are mainly associated with stress responses [52, 53], while some directly regulate flowering in multiple flowering pathways [22, 54, 55]. For example, IDD TFs belonging to the C2H2 family are involved in the regulation of flowering in sugar pathways [56, 57], while NF-YA, NF-YB, and NF-YC regulate flowering in response to light signals [58]. Additionally, SPL and AP2 TFs, which are the targets of two microRNAs (miRNA156 and miR172), mainly regulate phase transitions in age-related pathways [54, 59]. Therefore, these TFs that are differentially expressed between ‘Qinguan’ and ‘Nagafu No. 2’ buds may contribute to the floral induction associated with multiple biological processes.

### Flowering pathway genes that are differentially expressed between profusely flowering ‘Qinguan’ and weakly flowering ‘Nagafu No. 2’

To fully characterize the floral trait differences between the profusely flowering ‘Qinguan’ and weakly flowering ‘Nagafu No. 2’ apple varieties, we analysed approximately 190 flowering genes using genetic linkage maps (Fig. 11). Several studies have confirmed that key flowering genes directly control floral induction via multiple pathways [49, 60]. Consistent with these studies, we observed that key flowering genes, including *FT, FD*, and *LEAFY*, were more highly expressed in ‘Qinguan’ buds than in ‘Nagafu No. 2’ buds (Figs. 11 and 12 and Additional file 2). Additionally, *SOC1* expression is up-regulated by SPL9 and miR156 in the age-dependent flowering pathway [61]. We observed higher expression levels of *SPL9* in ‘Qinguan’ buds during floral induction (Figs. 11 and 12), which likely contributes to the higher flowering rate of this variety. Other genes, including *TFL1* and the negative regulator *SVP*, were expressed at lower levels in ‘Qinguan’ buds than in ‘Nagafu No. 2’ buds (Figs. 11 and 12). The protein encoded by *SVP* forms a floral repressor complex and binds to the *SOC1* promoter to supress expression [6, 62], while TFL1 is a floral repressor that inhibits flowering by regulating the expression of flowering genes such as *AP1*, *LEAFY*, and *FT* [63]. Thus, our results suggest that the differences in floral characteristics between the two analysed apple varieties are closely related to flowering gene expression levels.

## Conclusions

We applied RNA-seq data to compare gene expression patterns between the buds of profusely flowering ‘Qinguan’ and weakly flowering ‘Nagafu No. 2’ apple varieties during growth and floral induction. We observed that a complex genetic network involving carbon, fatty acid, lipid, and hormone-associated signalling regulatory mechanisms mediates apple tree floral induction. We also completed a qRT-PCR assay to analyse sugar-, hormone-, and flowering-related gene expression patterns in the buds of both apple varieties. Our findings may be useful for elucidating the molecular regulatory mechanisms underlying floral induction in apple trees.

## Methods

### Plant materials and sample collection

On 7 March 2008, 6-year-old trees of profusely flowering ‘Qinguan’ and weakly flowering ‘Nagafu No. 2’ apple varieties grafted on M.26 rootstocks were planted in the Apple Demonstration Nursery of Yangling Modern Agriculture Technology Park (Northwest Agriculture & Forestry University), Shaanxi, China (34°52′N, 108°7′E). Buds on the top spurs of ‘Qinguan’ and ‘Nagafu No. 2’ trees were collected during the early (ES; 5 May 2013), middle (MS; 1 June 2013), and late (LS; 25 June 2013) stages of flower bud physiological differentiation. For each variety, bud samples from 48 trees were combined. The bud samples were immediately placed in liquid nitrogen and stored at − 80 °C until used for the subsequent extraction and analysis of sugars and hormones as well as an RNA extraction and RNA-seq library construction. Total RNA was isolated from each sample using a modified version of a published method [2, 59].

### Analysis of bud break and flowering rates, dynamic changes in shoot and bud growth, and shoot types

From 2013 to 2015, bud break and flowering rates as well as the proportion of shoot types, including spurs (< 5 cm), intermediate shoots (5–15 cm) and long shoots (> 15 cm), were calculated for ‘Qinguan’ and ‘Nagafu No. 2’ (six trees each). The methods used to calculate flowering and bud break rates were as previously described [25, 64]. Shoot and bud growth and shoot types were measured and calculated using a tapeline and a digital-display Vernier calliper.

### Measurements of sugar and hormone contents

Sugars (i.e., sucrose, glucose, fructose, and sorbitol) and starch were extracted from approximately 0.3-mg (dry weight) samples of buds collected from ‘Qinguan’ and ‘Nagafu No. 2’ trees during the ES (5 May 2013), MS (1 June 2013), and LS (25 June 2013) flower bud differentiation stages. Extractions were performed as previously described [65]. Sugar contents were determined by high-performance liquid chromatography (Waters 2414 refractive index detector/Waters 1525 binary HPLC pump; Shaanxi, China) as previously described [66].

Phytohormones were extracted from approximately 0.5-mg (fresh weight) samples of buds collected from ‘Qinguan’ and ‘Nagafu No. 2’ trees at three developmental stages [i.e., ES (5 May 2013), MS (1 June 2013), and LS (25 June 2013) floral induction stages] [66]. Hormones were detected and analysed by high-performance liquid chromatography (Waters 2498 UV-Visible detector; Shaanxi, China) as previously described [67].

### Library construction and RNA deep sequencing

Six independent RNA-seq libraries from three ‘Qinguan’ bud samples [early stage (QE: 5 May 2013), middle stage (QM: 1 June 2013), and late stage (QL: 25 June 2013)] and three ‘Nagafu No. 2’ bud samples [early stage (FE: 5 May 2013), middle stage (FM: 1 June 2013), and late stage (FL: 25 June 2013)] during floral induction were constructed and sequenced on an Illumina Genome Analyzer by the Biomarker Biotechnology Corporation (Beijing, China). One RNA-seq library from each bud sample was construction and the RNA-seq library construction was performed according to previously described methods [2]. For each RNA-seq sample, transcript abundance was calculated based on the ratio of fragments per kilobase of transcript per million mapped read (FPKM) values [68]. Differentially expressed genes were detected as previously described [69]. A GO analysis of each gene to identify enriched biological processes, molecular functions, and cellular components was completed using *p* < 0.05 as the threshold for significant enrichment (http://www.geneontology.org/) [70]. The KEGG database was analysed to detect significantly enriched pathways based on a corrected *p*-value < 0.05 (https://www.genome.jp/kegg/pathway.html). For convenience, the prefix ‘MDP0000’ in each original gene ID (e.g., MDP0000600078) was abbreviated to ‘M’ (e.g., M600078).

### Venn diagrams of differentially expressed genes and analyses of their expression profiles

Venn diagrams of DEGs from the bud libraries of ‘Qinguan’ (QE, QM, and QL) and ‘Nagafu No. 2’ (FE, FM, and FL) were analysed [71, 72] using VENNTURE software (https://www.nia.nih.gov/research/resource/vennture). Additionally, a cluster analysis was performed and hierarchical clustering heat maps were generated with MultiExperiment Viewer 4.2 software (MEV4.2) (http://mev.tm4.org/) using the FPKM values of each gene.

### Validation of DEGs in a qRT-PCR assay

The relative expression levels of carbohydrate-, hormone-, and flowering-related genes in ‘Qinguan’ (QE, QM, and QL) and ‘Nagafu No. 2’ (FE, FM, and FL) buds during floral induction were detected by qRT-PCR using a previously described method [2]. Details regarding the qRT-PCR primers are provided in Additional file 1: Table S3.

### Statistical analysis

To assess differences in morphological (i.e., flowering rates, bud growth, and shoot length) and physiological (i.e., sugar and hormone contents) indicators and qRT-PCR data between ‘Qinguan’ and ‘Nagafu No 2’, a one-way analysis of variance with Tukey–Kramer multiple comparison tests was performed using DPS software version 7.0 (Zhejiang University, Hangzhou, China).

## Additional files

Additional file 1: **Table S1.** Summary of the sequencing data for the clean reads in each sample. **Table S2.** Summary of the sequencing data in each sample. **Table S3.** List of primers used in this study. **Figure S1.** Shoot length changes in ‘Qinguan’ and ‘Nagafu No. 2’ apple varieties on specific days after full bloom (DAFB). **Figure S2.** Proportion of spur, intermediate, and long shoots in ‘Qinguan’ and ‘Nagafu No. 2’ apple varieties. **Figure S3.** Sample read density on chromosomes. **Figure S4.** Number of differentially expressed genes in the buds of ‘Qinguan’ and ‘Nagafu No. 2’ apple varieties during floral induction. **Figure S5.** Number of differentially expressed cellular component genes in ‘Qinguan’ and ‘Nagafu No. 2’ during floral induction. (A) Up-regulated and (B) down-regulated differentially expressed genes (DEGs) in ‘Qinguan’ and ‘Nagafu No. 2’ buds. The seven types of DEGs (a-, b-, c-, d-, e-, f-, and g-type) are the same as those in the cluster analysis in Fig. 7. **Figure S6.** Number of differentially expressed molecular function genes in ‘Qinguan’ and ‘Nagafu No. 2’ during floral induction. **Figure S7.** Cluster analysis of differentially expressed *SAUR* family genes associated with the auxin response in ‘Qinguan’ and ‘Nagafu No. 2’ buds during floral induction. **Figure S8.** Cluster analysis of differentially expressed cell cycle-related genes in ‘Qinguan’ and ‘Nagafu No. 2’ buds during floral induction. **Figure S9.** Cluster analysis of differentially expressed transcription factor genes, grouped according to their respective families, in ‘Qinguan’ and ‘Nagafu No. 2’ buds during floral induction. **Figure S10.** Linear relationship between qRT-PCR data and RNA-seq data for related genes. (DOC 4856 kb)

Additional file 2: **Table S4.** Kyoto Encyclopedia of Genes and Genomes analysis of up-regulated genes, including the seven expression pattern types (a-, b-, c-, d-, e-, f-, and g-type) in ‘Qinguan’ buds relative to ‘Nagafu No. 2’ buds. The up-regulated genes were identified based on the Venn diagrams and cluster analysis in Fig. 5. **Table S5.** Kyoto Encyclopedia of Genes and Genomes analysis of down-regulated genes, including the seven expression pattern types (a-, b-, c-, d-, e-, f-, and g-type) in ‘Qinguan’ buds relative to ‘Nagafu No. 2’ buds. The down-regulated genes were identified based on the Venn diagrams and cluster analysis in Fig. 5. **Table S6.** Carbohydrate, fatty acid, and lipid pathways related to differentially expressed genes in ‘Qinguan’ and ‘Nagafu No. 2’ buds. The differentially expressed genes were detected based on the model in Fig. 7. **Table S7.** Hormone pathways related to differentially expressed genes in ‘Qinguan’ and ‘Nagafu No. 2’ buds. These hormone-related genes are mainly involved in hormone synthesis, signaling, and response pathways. The model and cluster analysis of these genes are presented in Figs. 8 and 9. **Table S8.** Detailed information of transcription factor family members in ‘Qinguan’ and ‘Nagafu No. 2’ buds. **Table S9.** Differentially expressed flowering-related genes in ‘Qinguan’ and ‘Nagafu No. 2’ buds. The flowering-related genes located on different chromosomes are presented in Fig. 11. (XLSX 153 kb)

## Abbreviations

ADPG: ADP-glucose
F-6-P: Fructose-6-phosphate
G-1-P: Glucose-1-phosphate
G-6-P: Glucose-6-phosphate
PGM: Phosphoglucomutase
S-6-P: Sorbitol-6-phosphate
S-6-P: Sucrose-6-phosphate
SUS: Sucrose synthase
TP: Triosephosphate
UDPG: UDP-glucose
