# Supplementary material for: Comparative RNA-sequencing-based transcriptome profiling of buds from profusely flowering ‘Qinguan’ and weakly flowering ‘Nagafu no. 2’ apple varieties reveals novel insights into the regulatory mechanisms underlying floral induction
Source: BMC Plant Biol. 2018 Dec 22;18:370. doi: 10.1186/s12870-018-1555-3 (PMC6303880; doi:10.1186/s12870-018-1555-3)
Supplement: Supplementary file 1 — Table S1. Summary of the sequencing data for the clean reads in each sample. Table S2. Summary of the sequencing data in each sample. Table S3. List of primers used in this study. Figure S1. Shoot length changes in ‘Qinguan’ and ‘Nagafu No. 2’ apple varieties on specific days after full bloom (DAFB). Figure S2. Proportion of spur, intermediate, and long shoots in ‘Qinguan’ and ‘Nagafu No. 2’ apple varieties. Figure S3. Sample read density on chromosomes. Figure S4. Number of differentially expressed genes in the buds of ‘Qinguan’ and ‘Nagafu No. 2’ apple varieties during floral induction. Figure S5. Number of differentially expressed cellular component genes in ‘Qinguan’ and ‘Nagafu No. 2’ during floral induction. (A) Up-regulated and (B) down-regulated differentially expressed genes (DEGs) in ‘Qinguan’ and ‘Nagafu No. 2’ buds. The seven types of DEGs (a-, b-, c-, d-, e-, f-, and g-type) are the same as those in the cluster analysis in Fig. 7. Figure S6. Number of differentially expressed molecular function genes in ‘Qinguan’ and ‘Nagafu No. 2’ during floral induction. Figure S7. Cluster analysis of differentially expressed SAUR family genes associated with the auxin response in ‘Qinguan’ and ‘Nagafu No. 2’ buds during floral induction. Figure S8. Cluster analysis of differentially expressed cell cycle-related genes in ‘Qinguan’ and ‘Nagafu No. 2’ buds during floral induction. Figure S9. Cluster analysis of differentially expressed transcription factor genes, grouped according to their respective families, in ‘Qinguan’ and ‘Nagafu No. 2’ buds during floral induction. Figure S10. Linear relationship between qRT-PCR data and RNA-seq data for related genes. (DOC 4856 kb) [file 12870_2018_1555_MOESM1_ESM.doc]

**Supplementary data**

Additional Supporting Information may be found in the online version of this article at the publisher’s website:

**Additional file 1**

**Table S1.** Summary of the sequencing data for the clean reads in each sample.

**Table S2.** Summary of the sequencing data in each sample.

**Table S3.** List of primers used in this study.

**Figure S1. Shoot length changes in ‘Qinguan’ and ‘Nagafu No. 2’ apple varieties on specific days after full bloom (DAFB).** (a) Shoot length. (b) Increase in shoot length per week. Data are presented as the mean ± standard error, *n* = 12. **p* < 0.05; ***p* < 0.01; ****p* < 0.001; ns, non-significant (*p* > 0.05).

**Figure S2. Proportion of spur, intermediate, and long shoots in ‘Qinguan’ and ‘Nagafu No. 2’ apple varieties.** Data are presented as the mean ± standard error, *n* = 12 in 2013, 2014, and 2015. **p* < 0.05; ***p* < 0.01; ****p* < 0.001; ns, non-significant (*p* > 0.05).

**Figure S3** **Sample read density on chromosomes.**

**Figure S4 Number of differentially expressed genes in the buds of ‘Qinguan’ and ‘Nagafu No. 2’ apple varieties during floral induction.** Early, middle, and late stages of flower bud differentiation are respectively denoted as FE, FM, and FL for ‘Nagafu No. 2’ and QE, QM, and QL for ‘Qinguan’.

**Figure S5 Number of differentially expressed cellular component genes in ‘Qinguan’ and ‘Nagafu No. 2’ during floral induction.** (A) Up-regulated and (B) down-regulated differentially expressed genes (DEGs) in ‘Qinguan’ and ‘Nagafu No. 2’ buds. The seven types of DEGs (a-, b-, c-, d-, e-, f-, and g-type) are the same as those in the cluster analysis in Figure 7.

**Figure S6 Number of differentially expressed molecular function genes in ‘Qinguan’ and ‘Nagafu No. 2’ during floral induction.** (A) Up-regulated and (B) down-regulated differentially expressed genes (DEGs) in ‘Qinguan’ and ‘Nagafu No. 2’ buds. The seven types of DEGs (a-, b-, c-, d-, e-, f-, and g-type) are the same as those in the cluster analysis in Figure 7.

**Figure S7 Cluster analysis of differentially expressed *SAUR* family genes associated with the auxin response in ‘Qinguan’ and ‘Nagafu No. 2’ buds during floral induction.**

**Figure S8 Cluster analysis of differentially expressed cell cycle-related genes in ‘Qinguan’ and ‘Nagafu No. 2’ buds during floral induction.**

**Figure S9. Cluster analysis of differentially expressed transcription factor genes, grouped according to their respective families, in ‘Qinguan’ and ‘Nagafu No. 2’ buds during floral induction.** See Supplementary Data 1 for more information regarding transcript abundance, differential expression, and gene annotations.

**Figure S10. Linear relationship between qRT-PCR data and RNA-seq data for related genes.**

**Table S1. Summary of the sequencing data for the clean reads in each sample.**

| **Sample** | **Total Reads** | **Total nucleotides(bp)** | **GC%** | **N%** | **Q20%** | **Q30%** |
| --- | --- | --- | --- | --- | --- | --- |
| **FE** | 20755348 | 4192245590 | 48.19 | 0.05 | 89.14 | 81.05 |
| **FM** | 22971288 | 4639814265 | 48.19 | 0.06 | 89.5 | 81.47 |
| **FL** | 19596243 | 3958059714 | 48.42 | 0.05 | 89.38 | 81.14 |
| **QE** | 21519449 | 4346474655 | 48.66 | 0.06 | 90.36 | 83.92 |
| **QM** | 18458264 | 3728234434 | 48.79 | 0.06 | 90.2 | 83.87 |
| **QL** | 16458329 | 3324307160 | 49.16 | 0.06 | 90.72 | 84.39 |

**Table S2. Summary of the sequencing data in each sample.**

| **Statistical content** | **FE** | | **FM** | | **FL** | | **QE** | | **QM** | | **QL** | |
| --- | --- | --- | --- | --- | --- | --- | --- | --- | --- | --- | --- | --- |
|  | **No.** | **%** | **No.** | **%** | **No.** | **%** | **No.** | **%** | **No.** | **%** | **No.** | **%** |
| **Total reads number** | 41510696 | 100% | 45942576 | 100% | 39192486 | 100% | 43038898 | 100 | 36916528 | 100 | 32916658 | 100 |
| **Mapped reads** | 21982043 | 52.96% | 24399499 | 53.11% | 20357313 | 51.94% | 23713245 | 0.551 | 20152276 | 0.5459 | 17658259 | 0.5365 |
| **Perfect Map** | 10156750 | 46.20% | 11411482 | 46.77% | 9675117 | 47.53% | 11686860 | 0.4928 | 10086474 | 0.5005 | 8731736 | 0.4945 |
| **Mismatch(0)** | 14388874 | 65.46% | 15935689 | 65.31% | 13096117 | 64.33% | 16055399 | 0.6771 | 13645725 | 0.6771 | 11908316 | 0.6744 |
| **Mismatch(1)** | 3590917 | 16.34% | 4055833 | 16.62% | 3498850 | 17.19% | 3705717 | 0.1563 | 3205462 | 0.1591 | 2793912 | 0.1582 |
| **Mismatch(2)** | 2075796 | 9.44% | 2334066 | 9.57% | 2150699 | 10.56% | 2118466 | 0.0893 | 1794045 | 0.089 | 1579837 | 0.0895 |
| **Indel** | 4232124 | 19.25% | 4524207 | 18.54% | 3421000 | 16.80% | 4368539 | 0.1842 | 3559251 | 0.1766 | 3176580 | 0.1799 |
| **Mismatch+Indel** | 1926456 | 8.76% | 2073911 | 8.50% | 1611647 | 7.92% | 1833663 | 0.0773 | 1507044 | 0.0748 | 1376194 | 0.0779 |
| **Unique Map** | 21041981 | 95.72% | 23364180 | 95.76% | 19327880 | 94.94% | 22747433 | 0.9593 | 19340465 | 0.9597 | 16893182 | 0.9567 |
| **Multiple Map** | 940062 | 4.28% | 1035319 | 4.24% | 1029433 | 5.06% | 965812 | 0.0407 | 811811 | 0.0403 | 765077 | 0.0433 |
| **Pair Map** | 15203951 | 69.17% | 16897618 | 69.25% | 13871112 | 68.14% | 17035935 | 0.7184 | 14518154 | 0.7204 | 12622701 | 0.7148 |
| **Single Map** | 5470603 | 24.89% | 6059172 | 24.83% | 5266875 | 25.87% | 5257224 | 0.2217 | 4467862 | 0.2217 | 4005652 | 0.2268 |

Note: Total reads; Mapped reads; Unique Mapped reads; Multiple Mapped reads; INDEL

**Table S3. List of primers used in this study.**

| **Genes** | **Gene ID** | **Forward (5’ to 3’)** | **Reverse (5’ to 3’)** |
| --- | --- | --- | --- |
| *HXK1* | MDP0000309677 | CTGAAAGTGGTCGGGAGCAAAC | TGCACGAGTGGCAACTATGTCG |
| *SDH* | MDP0000305455 | TGGGAGTGAGGTCGAGGATT | ATTGTACCGGCCTTGTTTGC |
| *FRK* | MDP0000765663 | TCAGGATGAGGAGGGGCTACGAG | CTGCTTTAAGCACTGGAGCACAGC |
| *SPS1* | MDP0000174537 | CCAAGTCGTCGTTGTTGCTA | TCTGTCTAGAGGTAGCGAGG |
| *NINV2* | MDP0000133399 | GAGTTCCAGACAGGCATAAGGCT | CCATCCGTCTATCAATCATACAGG |
| *FT* | MDP0000132050 | GCCAGCGAGGTTTCAACTTCTT | TGCCGCAGTAGTTGCTGGAATA |
| *FD* | MDP0000169473 | AGTGACCAGACCAACCACAACA | ATTTGGGTGGTGGGATCAGTGA |
| *AFL1* | MDP0000186703 | AAGGAGAGGGGCGAAAATG | GATGGAGAGACGGGGATGAG |
| *SPL9* | MDP0000297978 | TCGGGTGTCGGGATGAA | GTCGTCGGAAAGTGATTGGAG |
| *FKY1* | MDP0000220523 | AAGGCGGCATCCTCTTGTAGAT | TGCCTATGATGTGAGTGACGGT |
| *COL2* | MDP0000298635 | GTTGTTGCTCAACCCGGTGAAA | ATTGTTGTTGCTGCTGCTGGT |
| *CRY1* | MDP0000229393 | TGGCTCATCTTGATTGCTCCCT | TGGCACCAGTGGAAACAACAAC |
| *TFL1* | MDP0000255437 | CTCTTCAAGCAGAAACGAAGACAA | GTAAACGGCAGCGACAGGA |
| *ELF3* | MDP0000127365 | TCAACTCCGATGACAGGCAACT | AAACGGGTCCAGCAAACCAATG |
| *TIR1* | MDP0000125975 | TGGTAGTCACCGACGAGACC | CAAGAGGCACAGAACGATTGAG |
| *SHY2* | MDP0000295589 | GGTGATTACTGTGAGAAGAAA | TCTCAACCTCTTGCACGAAA |
| *PAP2* | MDP0000174664 | AGAATTCCCAAGGGTCTGCTGG | GAAGGAACGGAAGCCATTGAAT |
| *ARF2* | MDP0000258032 | ATGACGTCATCGGAGGTTTCG | TTCCTCGCGTCGTTGTGGTC |
| *IPT3* | MDP0000013380 | TCGGTCCAAGTATGACTGTTGC | CCTCATCCACCATTCCGTTT |
| *GA20ox* | MDP0000280240 | CACAACCTCCCTCCCCAAAAAC | ATCGGGCCAGATGAACTGACTC |
| *NCED3* | MDP0000228070 | ACAAGACACCGCCACCTTTC | TTGGGATTTGGATTACAGAAGG |
| *ETR1* | MDP0000557234 | TTGGCCTGTGAAGAGCAGT | TGCAAACCATGTAGAGCCAT |
| *MdACT* | | TGACCGAATGAGCAAGGAAATTACT | TACTCAGCTTTGGCAATCCACATC |


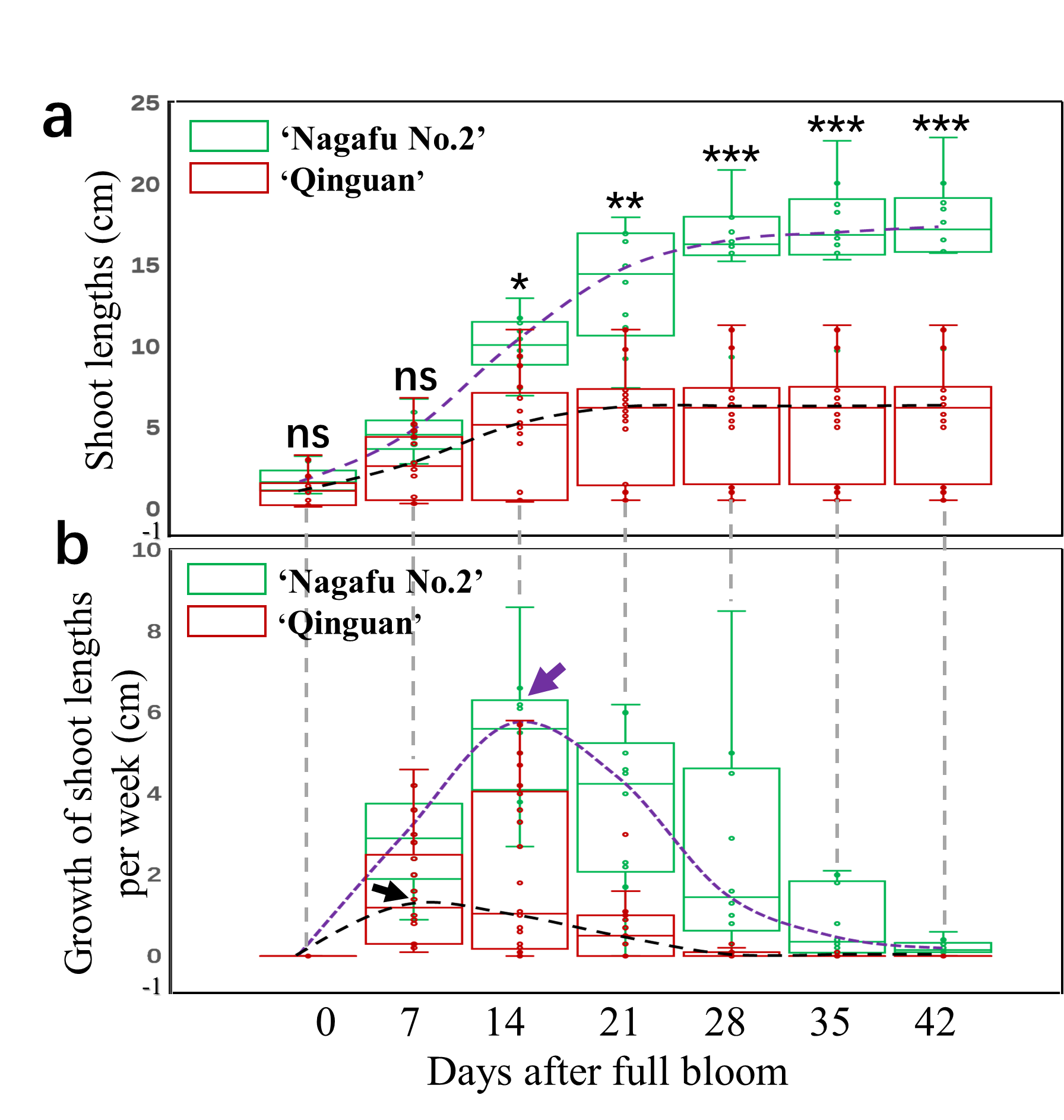


**Fig. S1. Shoot length changes in ‘Qinguan’ and ‘Nagafu No. 2’ apple varieties on specific days after full bloom (DAFB).** (a) Shoot length. (b) Increase in shoot length per week. Data are presented as the mean ± standard error, *n* = 12. **p* < 0.05; ***p* < 0.01; ****p* < 0.001; ns, non-significant (*p* > 0.05).


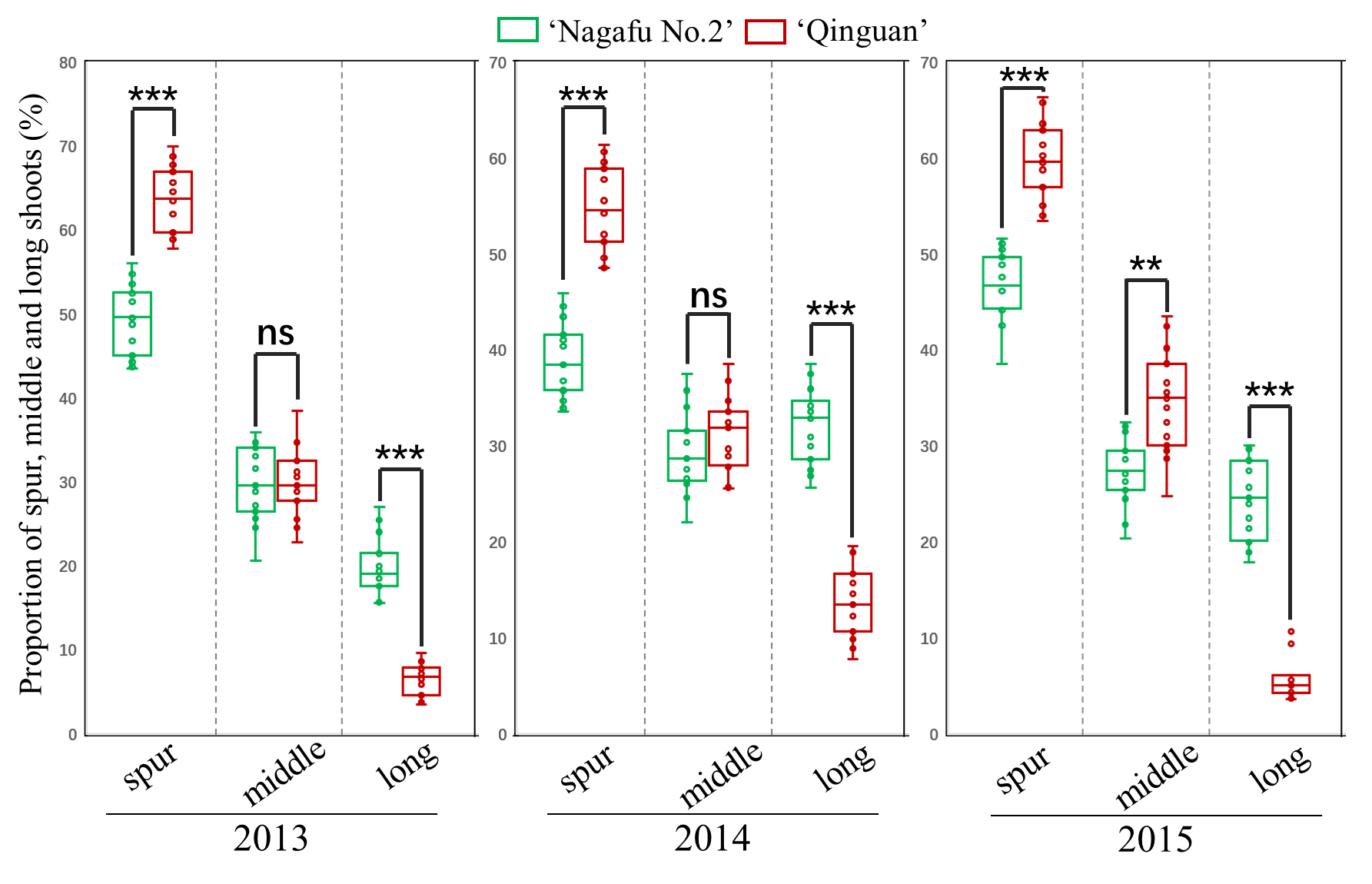


**Fig. S2. Proportion of spur, intermediate, and long shoots in ‘Qinguan’ and ‘Nagafu No. 2’ apple varieties.** Data are presented as the mean ± standard error, *n* = 12 in 2013, 2014, and 2015. **p* < 0.05; ***p* < 0.01; ****p* < 0.001; ns, non-significant (*p* > 0.05).


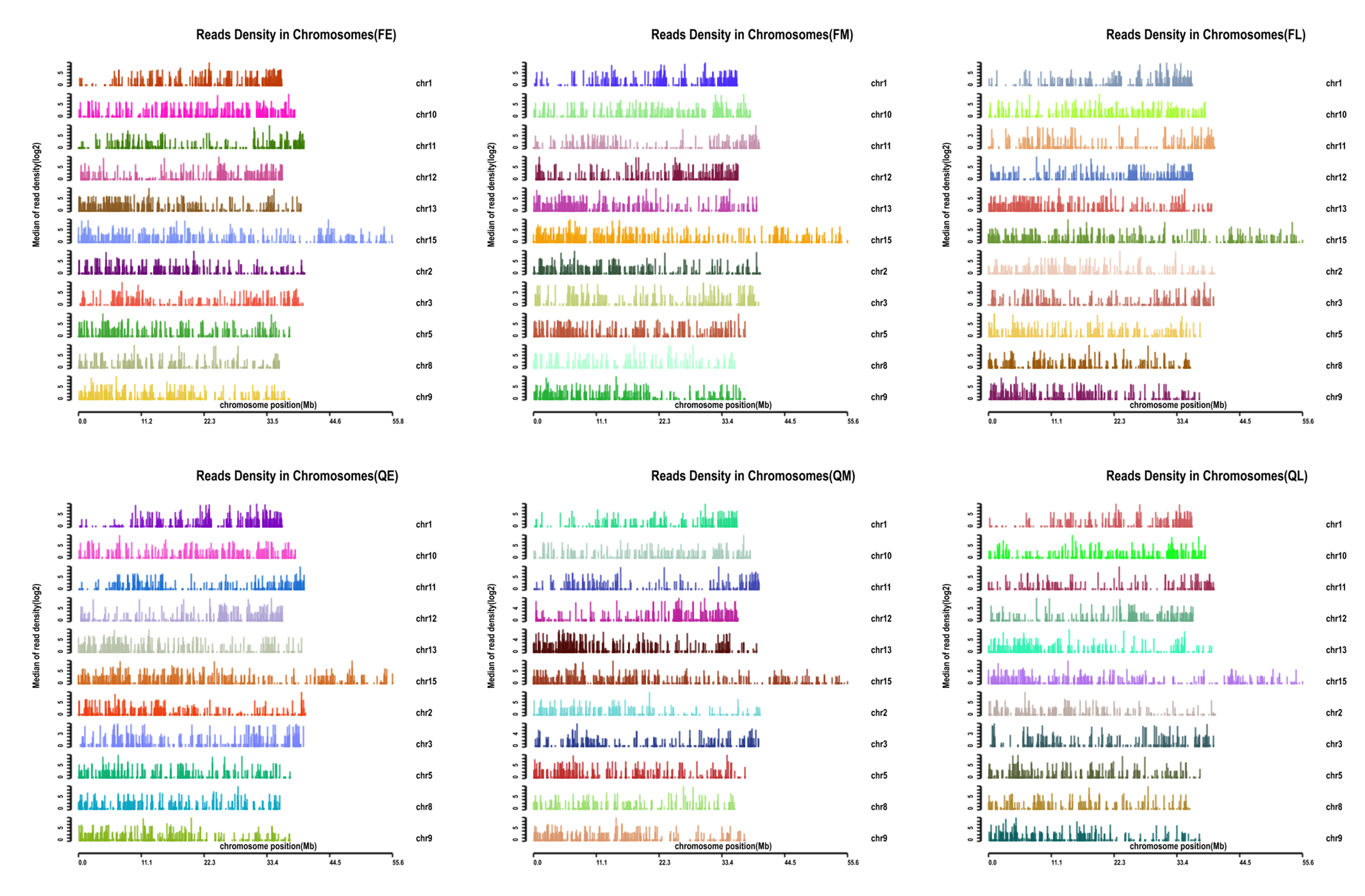


**Fig. S3.** **Sample read density on chromosomes.** Early, middle, and late stages of flower bud differentiation are respectively denoted as FE, FM, and FL for ‘Nagafu No. 2’ and QE, QM, and QL for ‘Qinguan’. Different colors mean read density in different chromosomes.


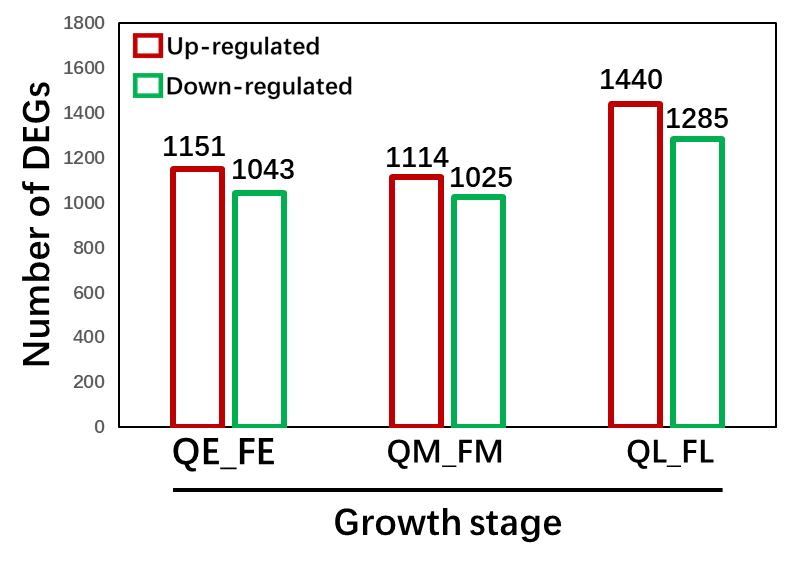


**Fig. S4. Number of differentially expressed genes in the buds of ‘Qinguan’ and ‘Nagafu No. 2’ apple varieties during floral induction.** Early, middle, and late stages of flower bud differentiation are respectively denoted as FE, FM, and FL for ‘Nagafu No. 2’ and QE, QM, and QL for ‘Qinguan’.


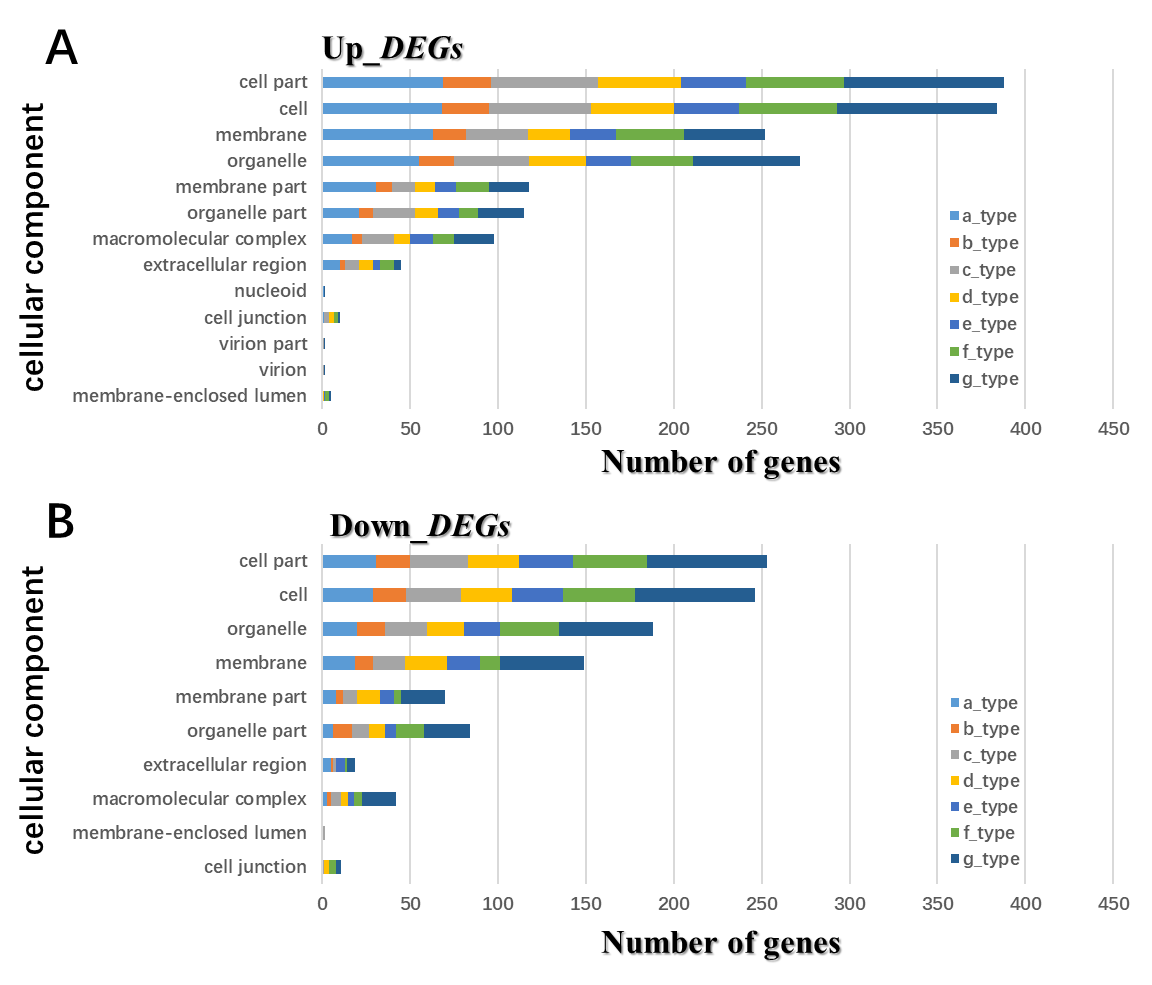


**Fig. S5. Number of differentially expressed cellular component genes in ‘Qinguan’ and ‘Nagafu No. 2’ during floral induction.** (A) Up-regulated and (B) down-regulated differentially expressed genes (DEGs) in ‘Qinguan’ and ‘Nagafu No. 2’ buds. The seven types of DEGs (a-, b-, c-, d-, e-, f-, and g-type) are the same as those in the cluster analysis in Figure 7.


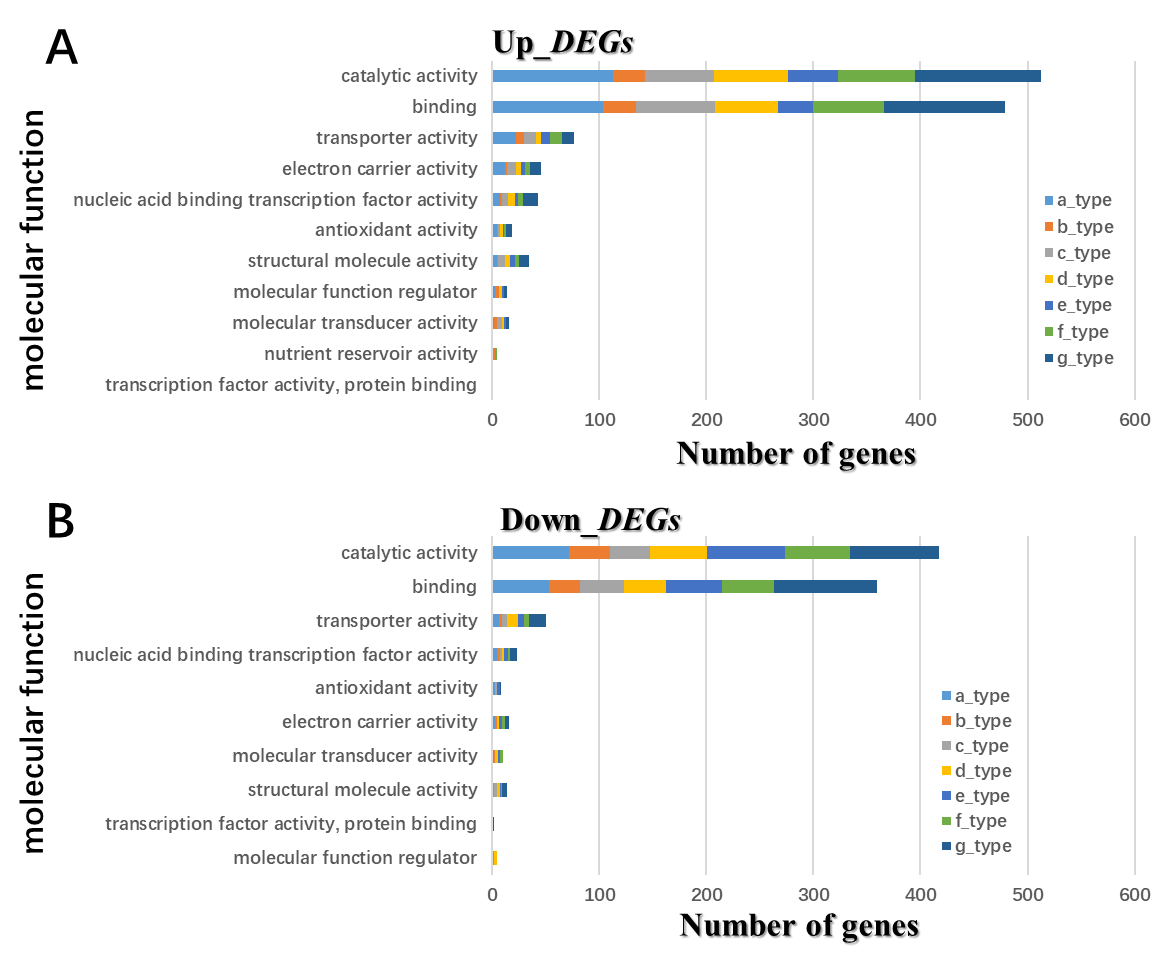


**Fig. S6. Number of differentially expressed molecular function genes in ‘Qinguan’ and ‘Nagafu No. 2’ during floral induction.** (A) Up-regulated and (B) down-regulated differentially expressed genes (DEGs) in ‘Qinguan’ and ‘Nagafu No. 2’ buds. The seven types of DEGs (a-, b-, c-, d-, e-, f-, and g-type) are the same as those in the cluster analysis in Figure 7.


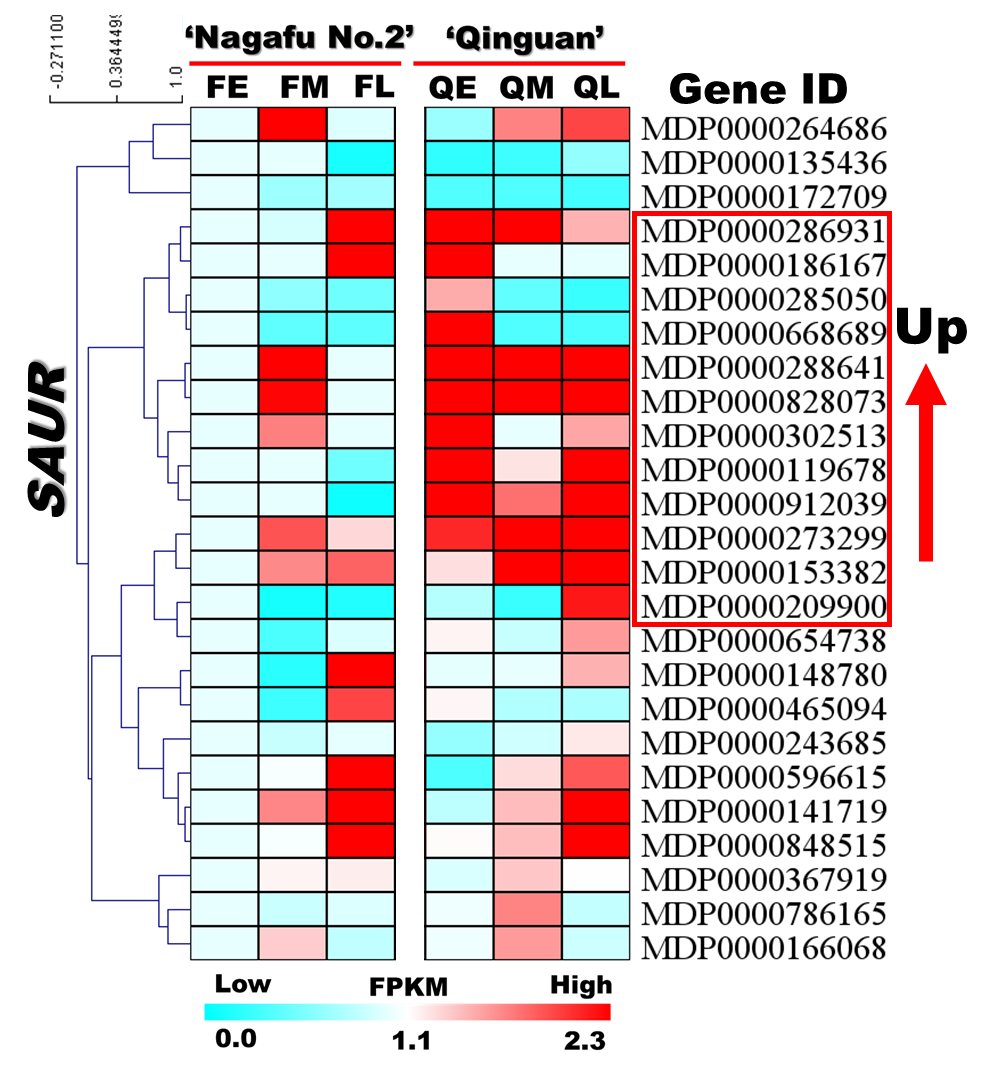


**Fig. S7. Cluster analysis of differentially expressed *SAUR* family genes associated with the auxin response in ‘Qinguan’ and ‘Nagafu No. 2’ buds during floral induction.**


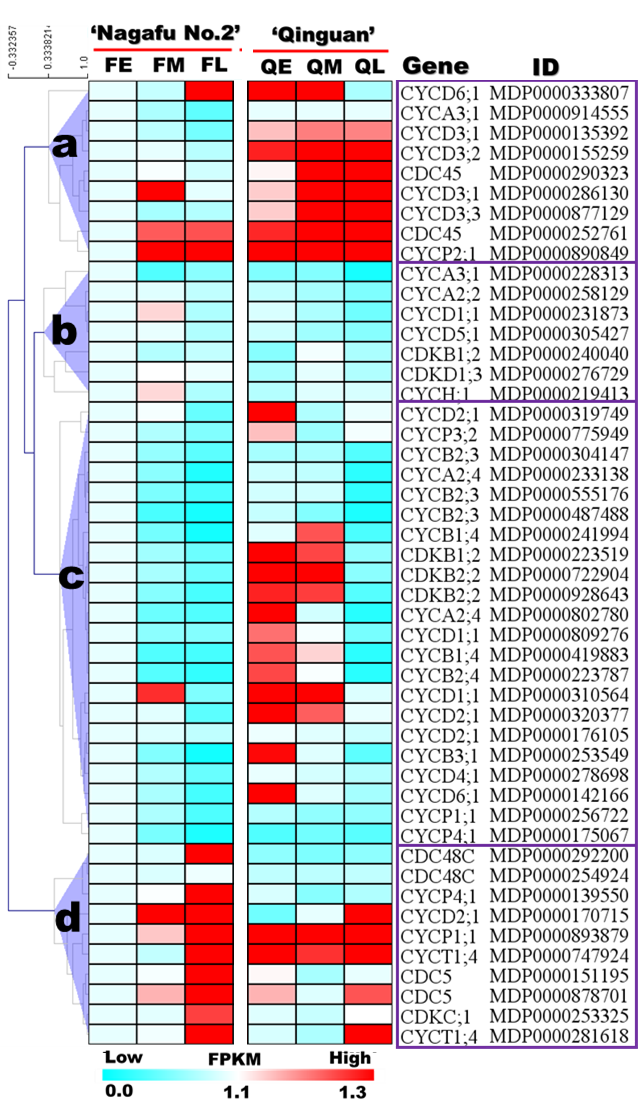


**Fig. S8. Cluster analysis of differentially expressed cell cycle-related genes in ‘Qinguan’ and ‘Nagafu No. 2’ buds during floral induction.**


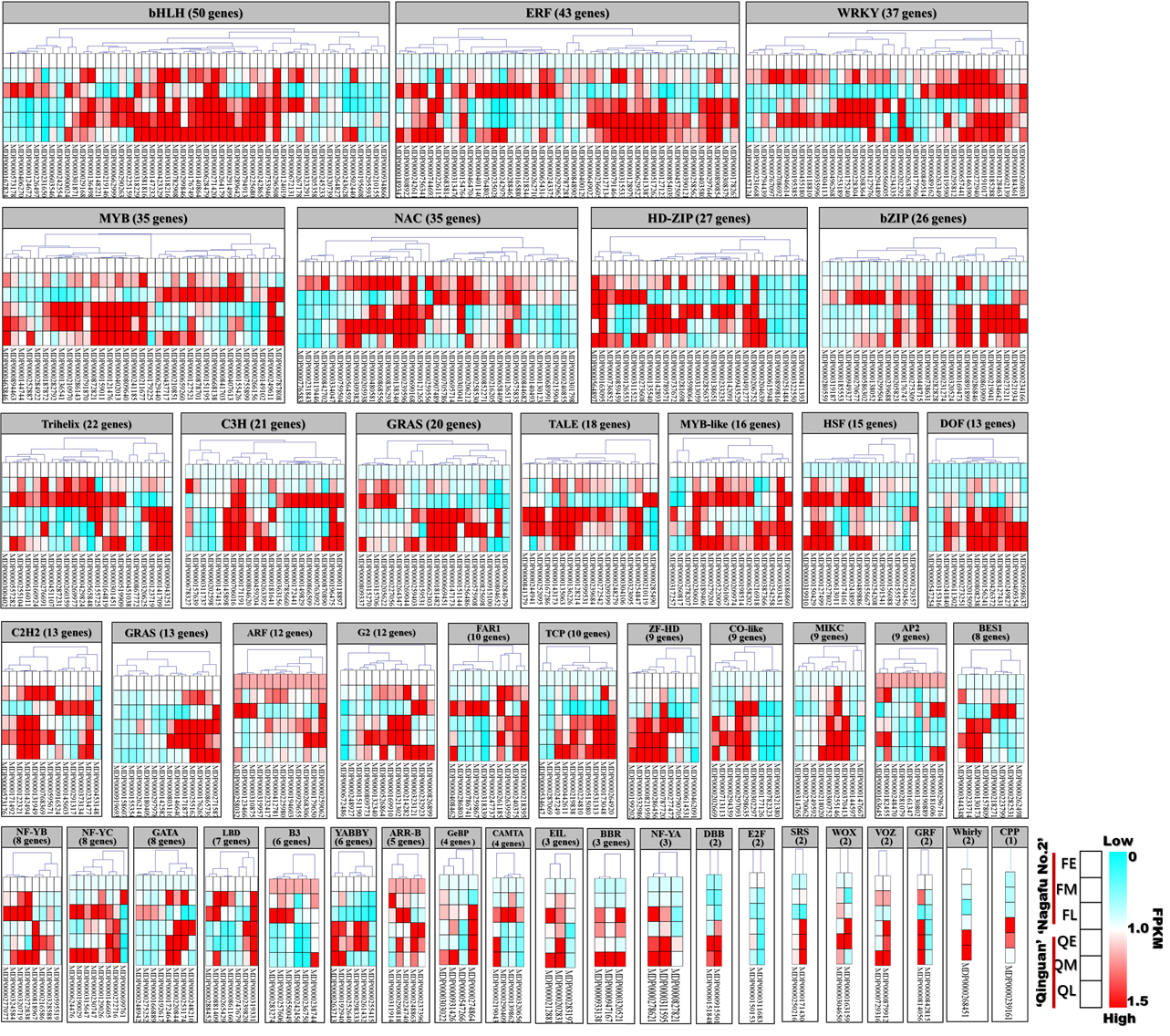


**Fig. S9. Cluster analysis of differentially expressed transcription factor genes, grouped according to their respective families, in ‘Qinguan’ and ‘Nagafu No. 2’ buds during floral induction.** See Supplementary Data 1 for more information regarding transcript abundance, differential expression, and gene annotations.


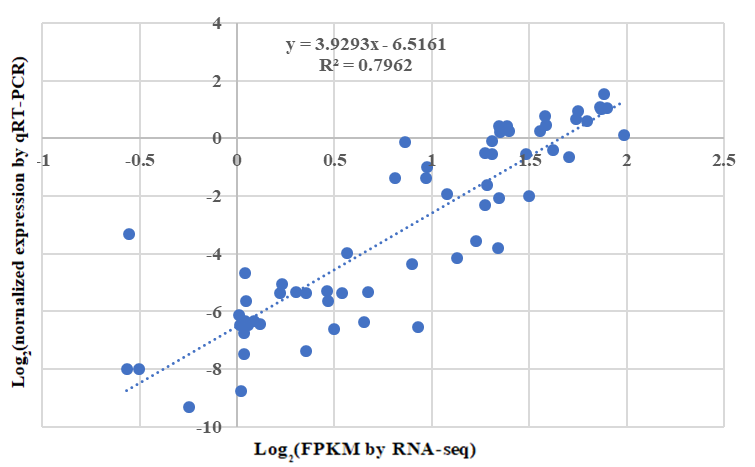


**Fig S10. Linear relationship between qRT-PCR data and RNA-seq data of related genes.**
